# Supplementary figures and images for: Rubicon prevents autophagic degradation of GATA4 to promote Sertoli cell function
Source: PLoS Genet. 2021 Aug 5;17(8):e1009688. doi: 10.1371/journal.pgen.1009688 (PMC8341604; doi:10.1371/journal.pgen.1009688)

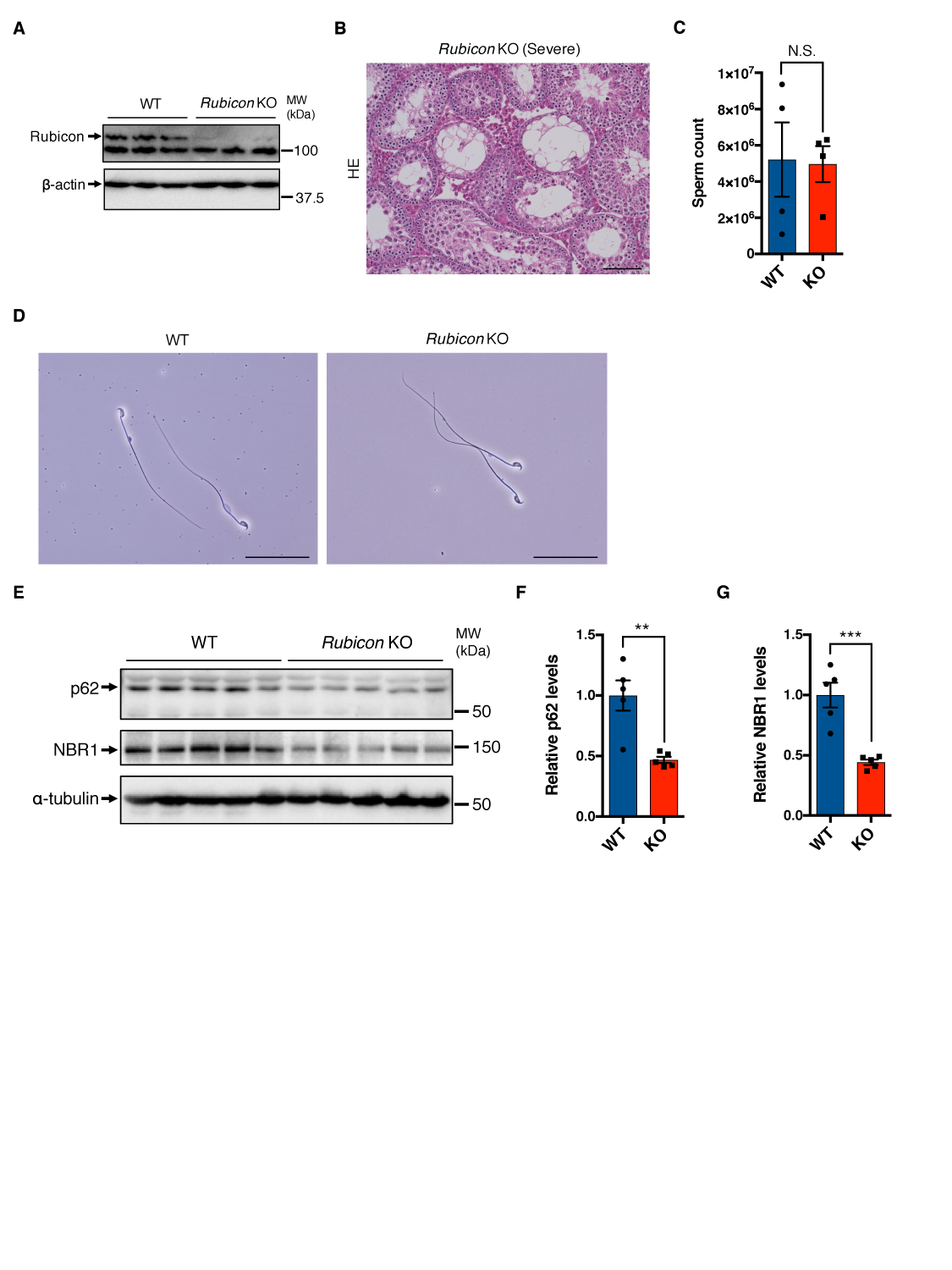

Supplement: S1 Fig — (A) Immunoblotting of Rubicon in testes from mice of the indicated genotypes. n = 3. (B) Representative images of H&E staining of testis section from Rubicon KO mouse with a severe defect. Scale bars, 100 μm. (C) Sperm counts from the cauda epididymis of WT or Rubicon KO mice. n = 4. (D) Representative images of spermatozoa from mice of the indicated genotypes. Scale bars, 50 μm. (E) Immunoblotting of the indicated proteins in testes from mice of the indicated genotypes. n = 5. (F and G) Quantification of relative p62 (F) and NBR1 (G) levels in (E). Error bars indicate means ± SEM. Data were analyzed by two-tailed Student’s t-test (C, F, G). *P < 0.05, **P < 0.01, ***P < 0.001. N.S., not significant. (TIF) [file pgen.1009688.s001.tif]

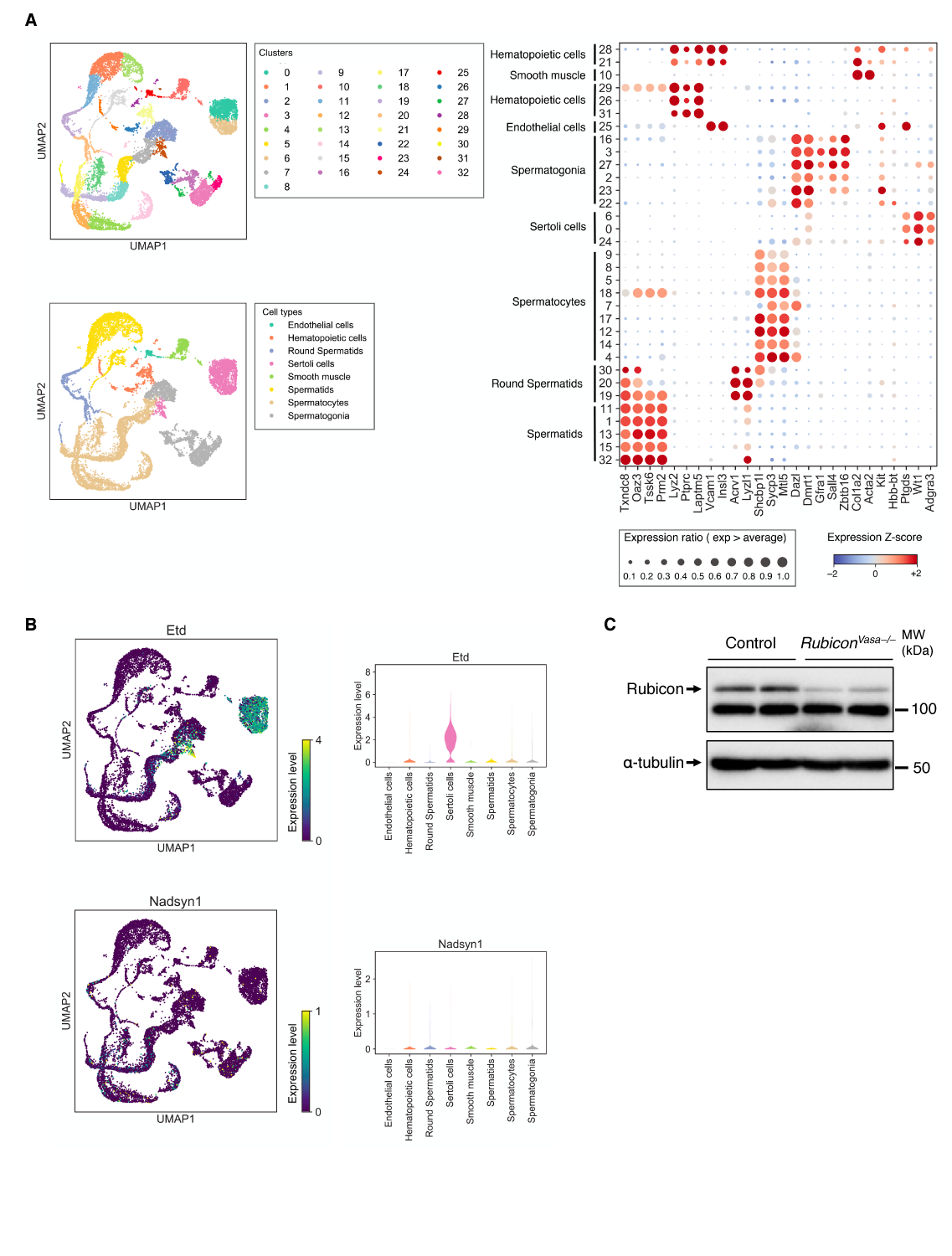

Supplement: S2 Fig — (A) Uniform Manifold Approximation and Projection (UMAP) plot representing 32 cell clusters from the published scRNA-seq data. Dotplot depicting selected marker genes in cell clusters. (B) UMAP and Violin plots of Etd and Nadsyn1 expression levels in eight testicular cell types. (C) Immunoblotting of Rubicon in testes from mice of the indicated genotypes. n = 3. (TIF) [file pgen.1009688.s002.tif]

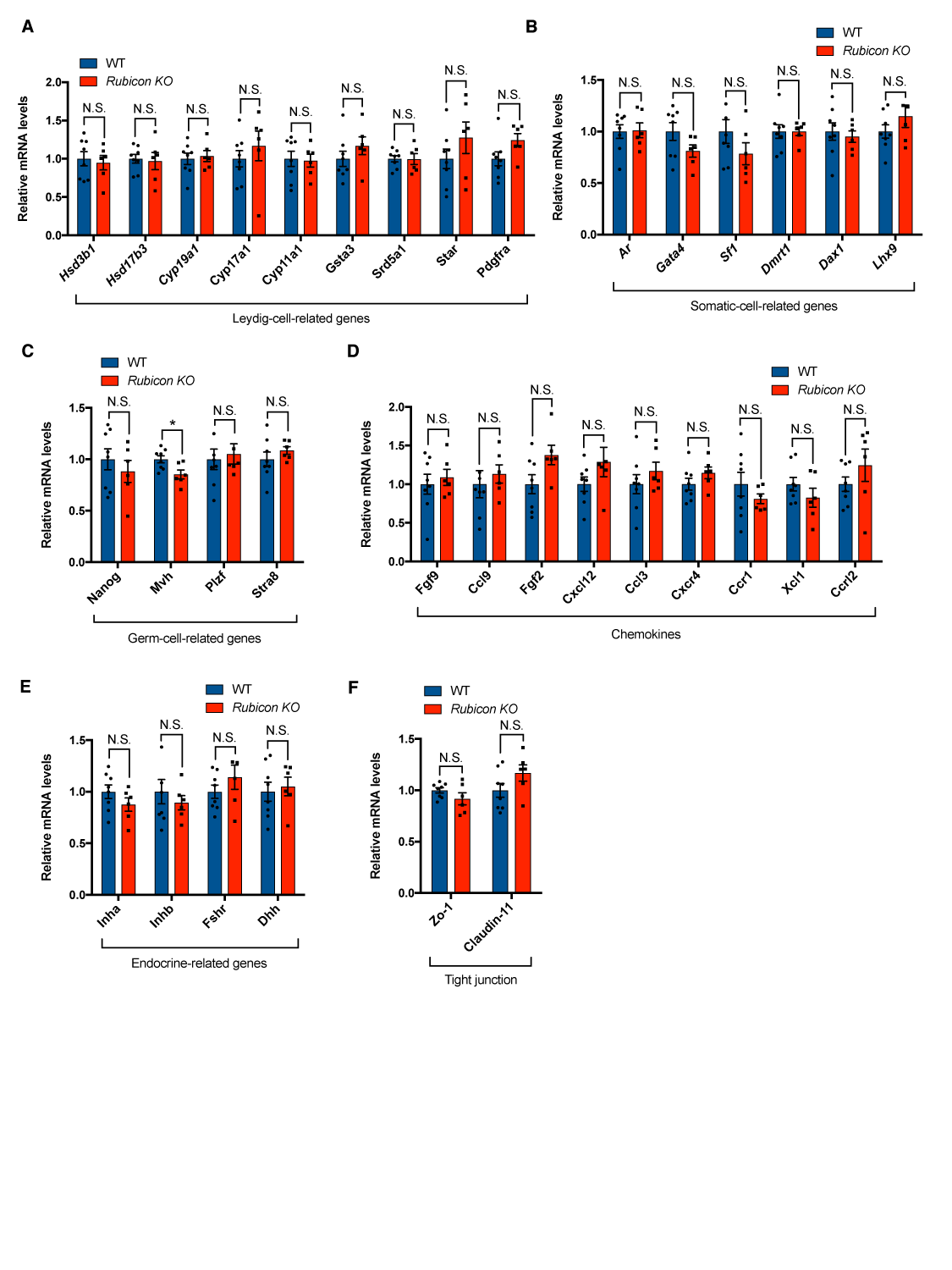

Supplement: S3 Fig — (A) Relative mRNA levels of Leydig-cell-related genes in testes from mice of the indicated genotypes. WT, n = 8; Rubicon KO, n = 6. (B) Relative mRNA levels of somatic-cell-related genes in testes from mice of the indicated genotypes. WT, n = 8; Rubicon KO, n = 6. (C) Relative mRNA levels of germ-cell-related genes in testes from mice of the indicated genotypes. WT, n = 8; Rubicon KO, n = 6. (D) Relative mRNA levels of chemokine genes in testes from mice of the indicated genotypes. WT, n = 8; Rubicon KO, n = 6. (E) Relative mRNA levels of endocrine-related genes in testes from mice of the indicated genotypes. WT, n = 8; Rubicon KO, n = 6. (F) Relative mRNA levels of tight junction genes in testes from mice of the indicated genotypes. WT, n = 8; Rubicon KO, n = 6. Error bars indicate means ± SEM. Data were analyzed by two-tailed Student’s t-test (A–F). N.S., not significant. (TIF) [file pgen.1009688.s003.tif]

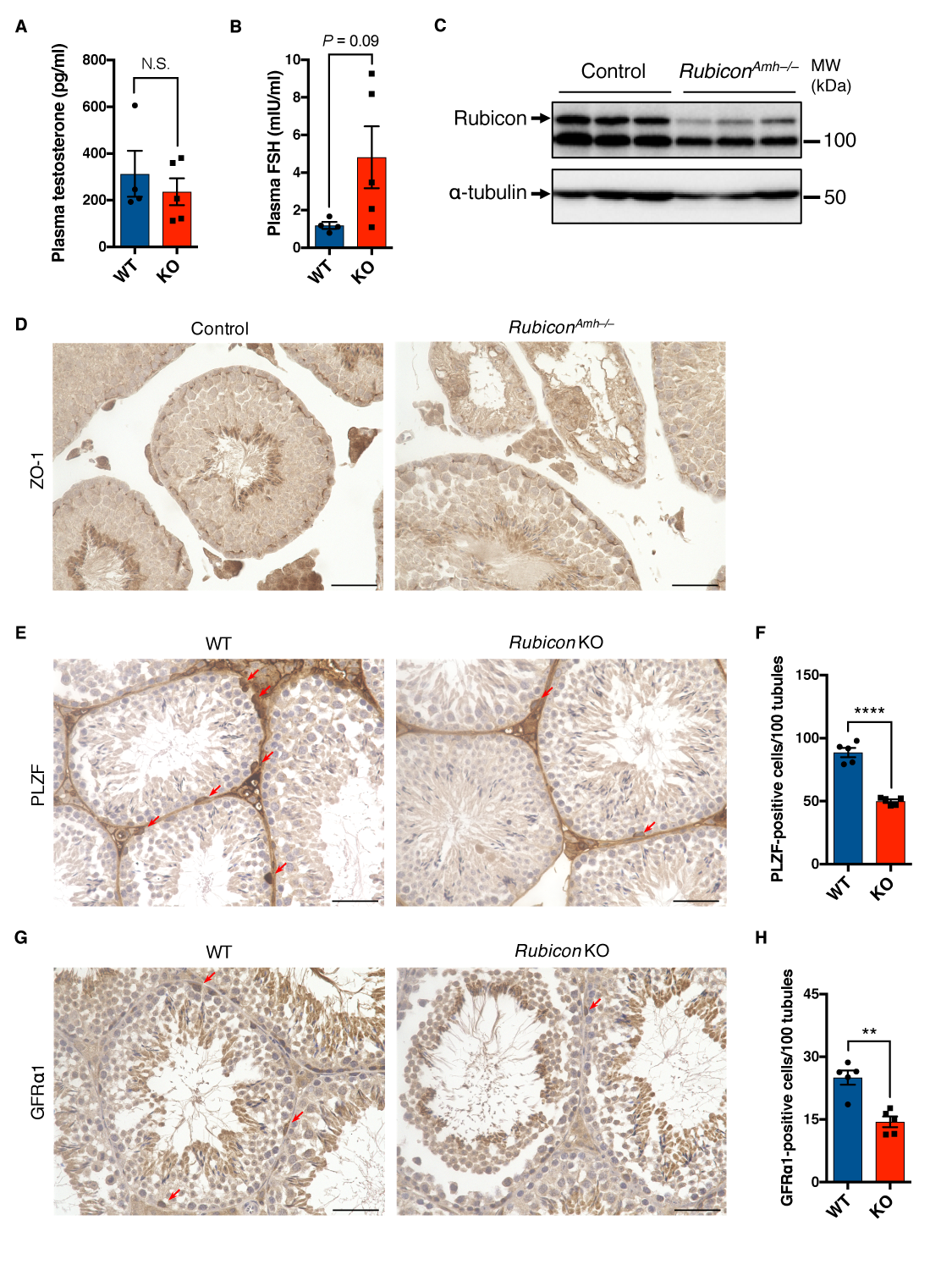

Supplement: S4 Fig — (A) Plasma testosterone levels in mice of the indicated genotypes. WT, n = 4; Rubicon KO, n = 5. (B) Plasma FSH levels in mice of the indicated genotypes. WT, n = 4; Rubicon KO, n = 5. (C) Immunoblotting of Rubicon in testes from mice of the indicated genotypes. n = 3. (D) Representative images of immunohistochemistry to detect ZO-1 in testis sections from mice of the indicated genotypes. Scale bars, 50 μm. Control, n = 8; RubiconAmh–/–, n = 6. (E) Representative images of immunohistochemistry to detect PLZF in testis sections from mice of the indicated genotypes. Red arrows indicate positive staining. Scale bars, 50 μm. n = 5. (F) Quantification of PLZF-positive cells in (E). (G) Representative images of immunohistochemistry to detect GFRα1 in testis sections from mice of the indicated genotypes. Red arrows indicate positive staining. Scale bars, 50 μm. n = 5. (H) Quantification of GFRα1-positive cells in (G). Error bars indicate means ± SEM. Data were analyzed by two-tailed Student’s t-test (A, B, F, H). *P < 0.05, **P < 0.01, ***P < 0.001, ****P < 0.0001. N.S., not significant. (TIF) [file pgen.1009688.s004.tif]

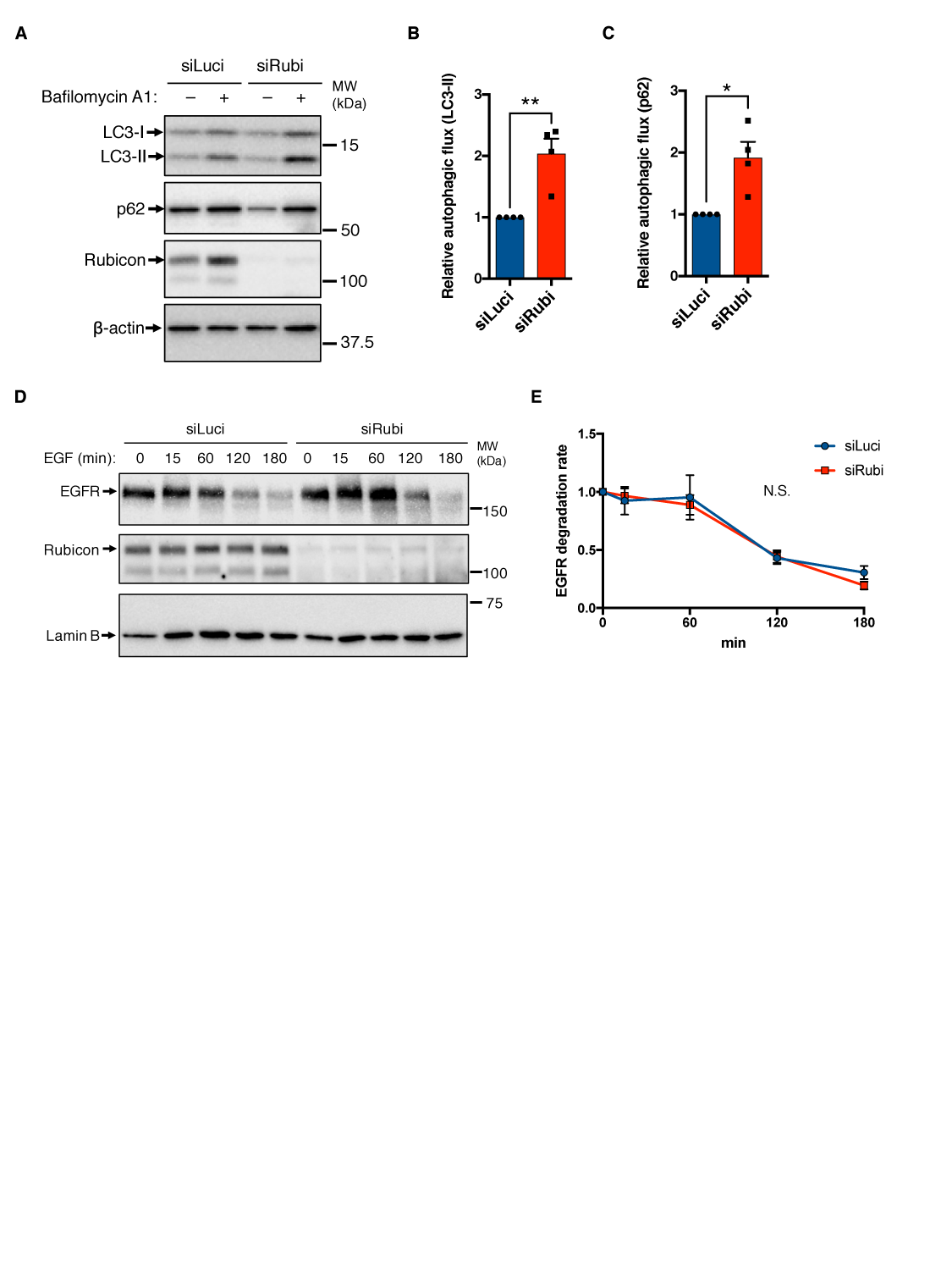

Supplement: S5 Fig — (A) Autophagic flux assay using LC3-II and p62 degradation in Luciferase or Rubicon knockdown 15P-1 cells. Knockdown was carried out for 48 h. n = 4. (B and C) Quantification of autophagic flux in (A) using LC3-II (B) and p62 (C). (D) EGFR degradation assay in Luciferase or Rubicon knockdown 15P-1 cells. Knockdown was carried out for 48 h. n = 4. (E) Quantification of the relative EGFR level in (D). Error bars indicate means ± SEM. Data were analyzed by two-tailed Student’s t-test (B, C), two-way ANOVA (E). *P < 0.05, **P < 0.01. N.S., not significant. (TIF) [file pgen.1009688.s005.tif]
