# Supplementary material for: Rubicon prevents autophagic degradation of GATA4 to promote Sertoli cell function
Source: PLoS Genet. 2021 Aug 5;17(8):e1009688. doi: 10.1371/journal.pgen.1009688 (PMC8341604; doi:10.1371/journal.pgen.1009688)

Point-by-point response

Reviewer #1**:**

General comments

The MS described that a negative regulator of autophagy, Rubicon, is required for spermatogenesis. And it mainly worked in Sertoli cells, the disruption of this gene might promote autophagic degradation of GATA4, which is a transcription factor that is essential for Sertoli cell function. This is an interesting story which should be considered to be published in the Journal of PLOS Genetics. However, before its publication, the following questions need to be addressed.

We appreciate your insightful suggestions that have greatly helped us to improve our study. Following is the detailed response to your comments. We have highlighted the changes in the revised manuscript by using blue color.

Comment-1

Is GATA4 the key substrate which need to be eliminated by Rubicon or autophagy? In that case, the overexpression of this gene should at least partially rescue the spermatogenic defects of Rubicon KO mice. If this point were difficult to be directly tested, some autophagy inhibitors such as 3-MA, Chloroquine and bafilomycin A1 should be tested to see if any of them could rescue the male reproductive defect of Rubicon KO mice, meanwhile, the GATA 4 level should be measured.

Reply-1

We totally agree with your idea. It is to be determined whether GATA4 is the key substrate in *Rubicon* KO mice. However, as clearly demonsrated by previous studies, autophagy deficiency in germ cells or Sertoli cells causes a severe defect in spermatogenesis (H Wang *et al*., ***Cell Research*** 2014; Y Shang *et al*., ***Autophagy*** 2016; C Liu *et al*., ***Autophagy*** 2016). Therefore, it is difficult to see whether chronic autophagy inhibitor treatment improves the defects in systemic *Rubicon* KO mice. Instead of this, we treated the KO mice with chloroquine for eight hours, and found that this treatment completely canceled the reduction of GATA4 in the testis from the Rubicon KO mice (Figs 4F and 4G). And importantly, bafilomycin A1 specifically increases the levels of GATA4 in 15P-1 Sertoli cells (Figs 5C–5E). The levels of GATA4, but neither SOX9, WT1 nor AR, were significantly reduced under starvation treatment (Figs 6H–6J). Together with the previous report that showed the requirement of GATA4 in Sertoli cells (SR Chen *et al*., ***Oncotarget*** 2015), this data strongly suggests that GATA4 is the key autophagic substrate in the testis of *Rubicon* KO mice. We added the associated references to the revised manuscript (Page 5, Line 88, 89).

Fig 4F

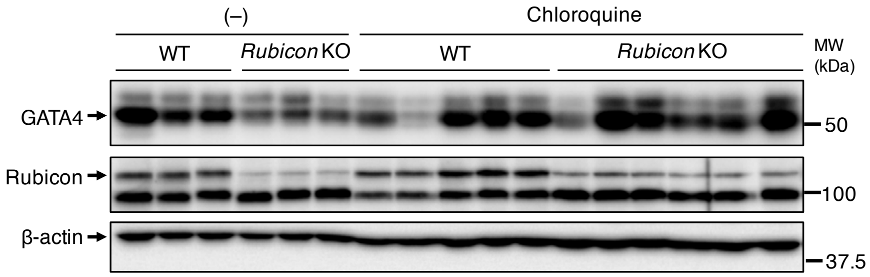

Fig 4G

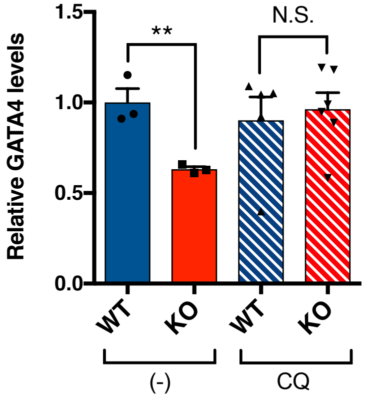

Fig 5C

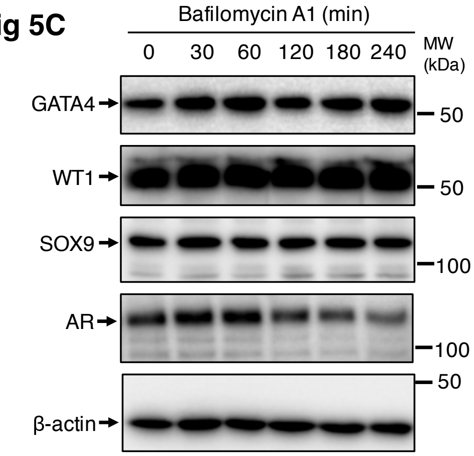

Fig 5D

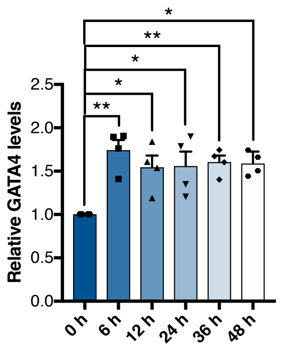

Fig 5E

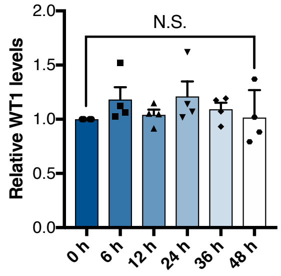

Fig 6H

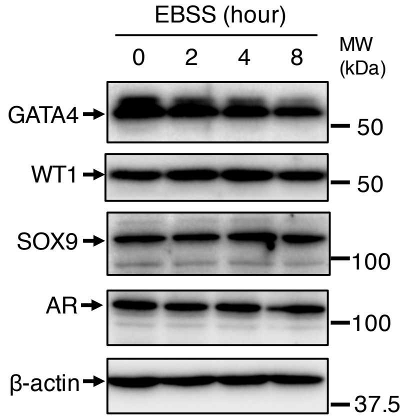

Fig 6I

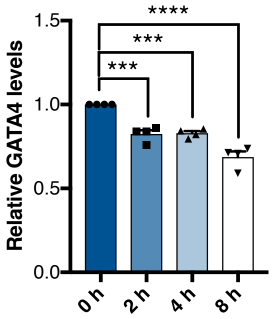

Fig 6J

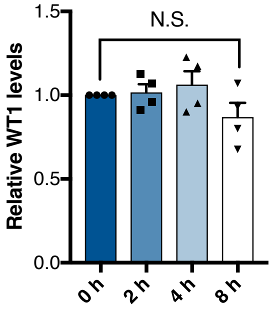

Comment-2

There is giant gap between androgen and Rubicon/autophagy. The data in Figure 5 only show some relationship between androgen and Rubicon/autophagy which is still far from conclusive. Apply the same method to mouse should get much more information about their relationship, and the data should be solid than that of those clinical results.

Reply-2

According to your constructive suggestion, we injected androgen antagonists into mice. Androgen antagonist treatment significantly reduced the levels of Rubicon and GATA4 in testis (Figs 7A–7C). This is consistent with the clinical results showing that anti-androgen therapy decreases the levels of Rubicon and GATA4. We redid and quantified the immunostaining of the clinical samples for GATA4 (Figs 7E–7G). We had found that androgen antagonist treatment leads to a reduction of Rubicon and GATA4 levels in 15P-1 Sertoli cells (Figs 6A–6C). These observations indicate that androgen negatively regulates testicular levels of Rubicon and GATA4.

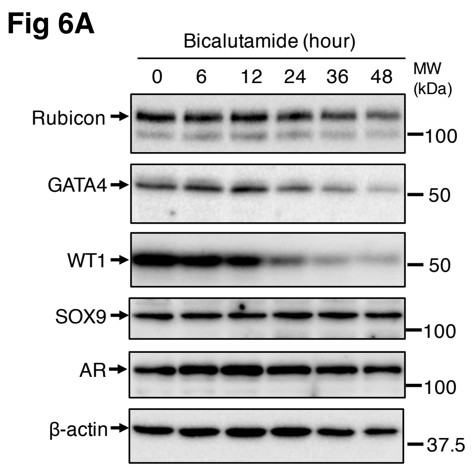

Fig 7A

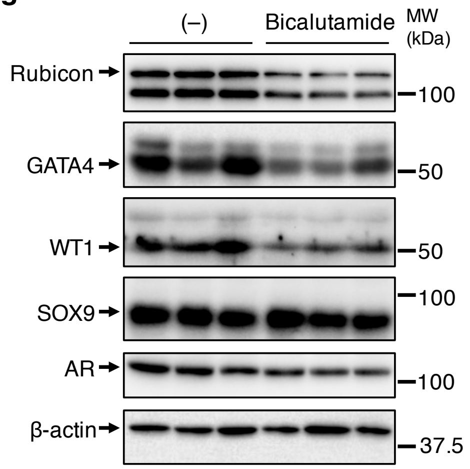

Fig 7B

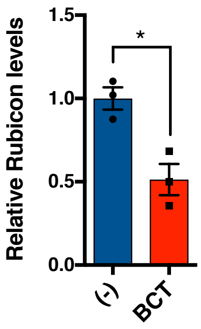

Fig 7C

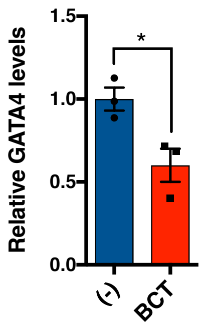

Fig 7G

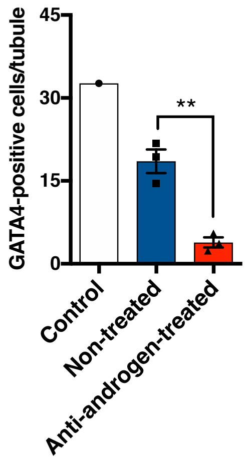

Fig 7E

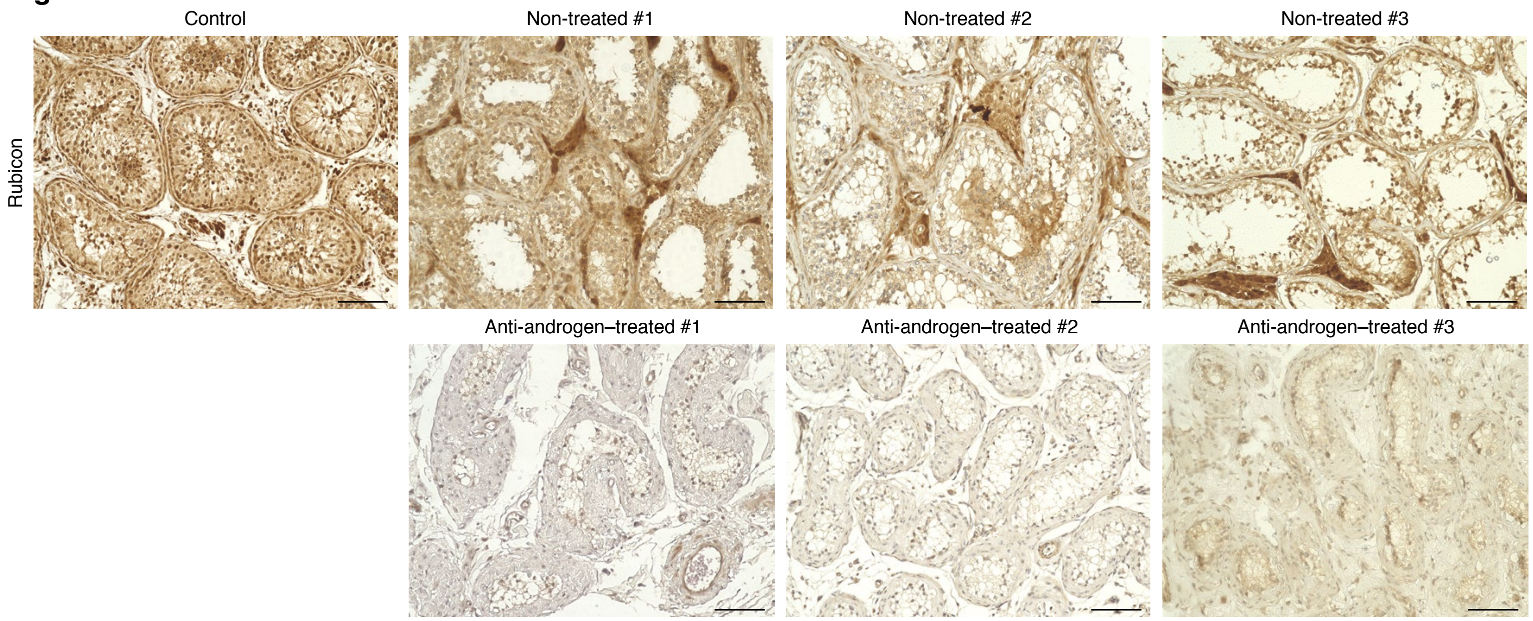

Fig 7F

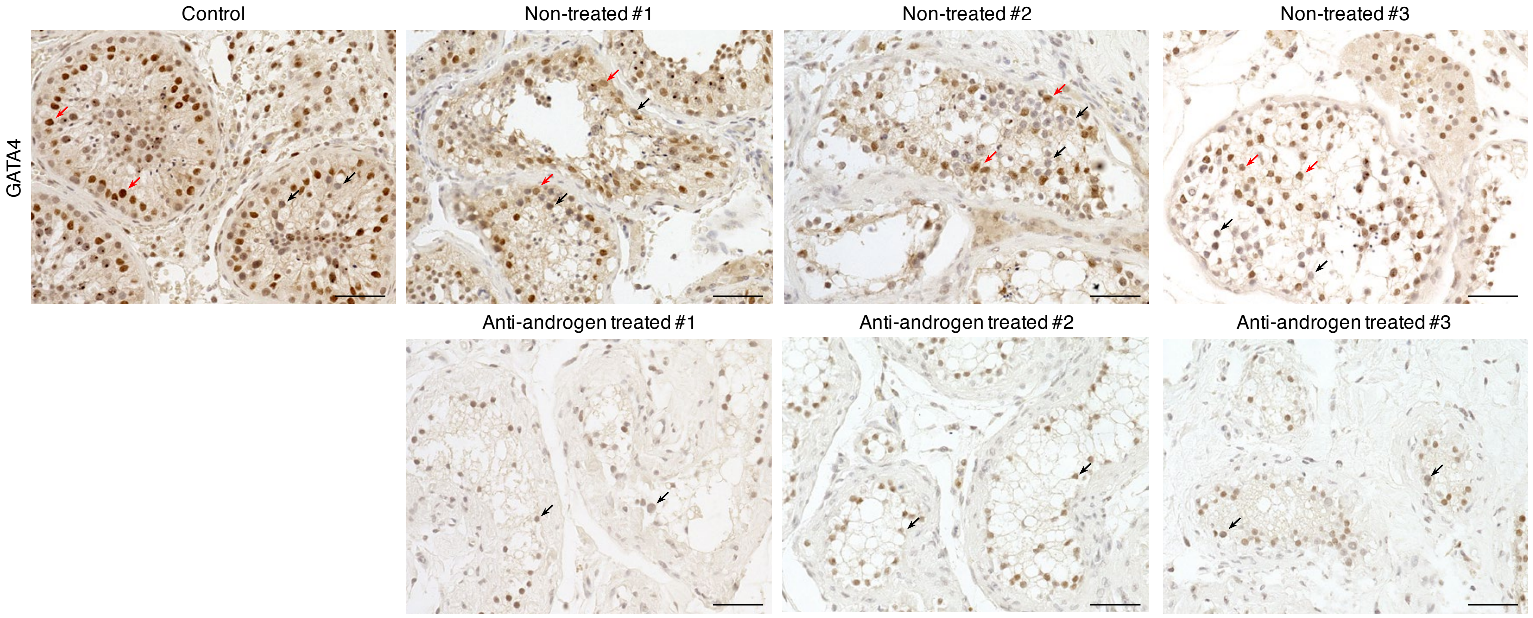

Comment-3

A lot of stresses could trigger autophagy, thus downregulate GATA4 level. Except androgen antagonist, other kind of physiological stimulators also should be considered.

Reply-3

Thank you for your comment. This is very informative. As also shown in Reply-1, to induce autophagy, we subjected 15P-1 cells to a starvation medium EBSS. The levels of GATA4 were significantly reduced (Figs 6H–6J), suggesting that GATA4 is an autophagic substrate and thus is downregulated in autophagy-stimulated conditions.

Fig 6H **Fig 6I** **Fig 6J**

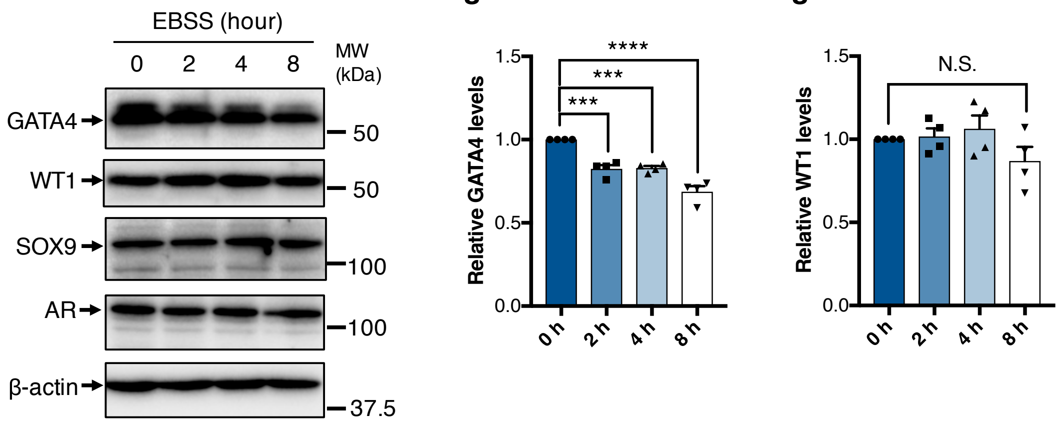

Comment-4

Because the Sertoli cells can be cultured in vitro, the key point of this MS should be directly tested in Sertoli cells but not only the cell line.

Reply-4

This is very important for our study. Because we could not establish a primary Sertoli cell culture system, we examined the levels of GATA4 in Sertoli cells *in vivo* by using SOX9 as a Sertoli cell marker. Consistent with the cell line data, Sertoli-cell-specific *Rubicon* KO mice exhibited a substantial reduction of the GATA4-positive rate in SOX9-positive Sertoli cells (Figs 4C and 4E). This data strongly suggests that Rubicon maintains GATA4 levels in Sertoli cells *in vivo*. We also found that SOX9-positive cells were significantly reduced in the knockout mice (Fig 4D), indicating that Rubicon is required for the maintenance of Sertoli cell number.

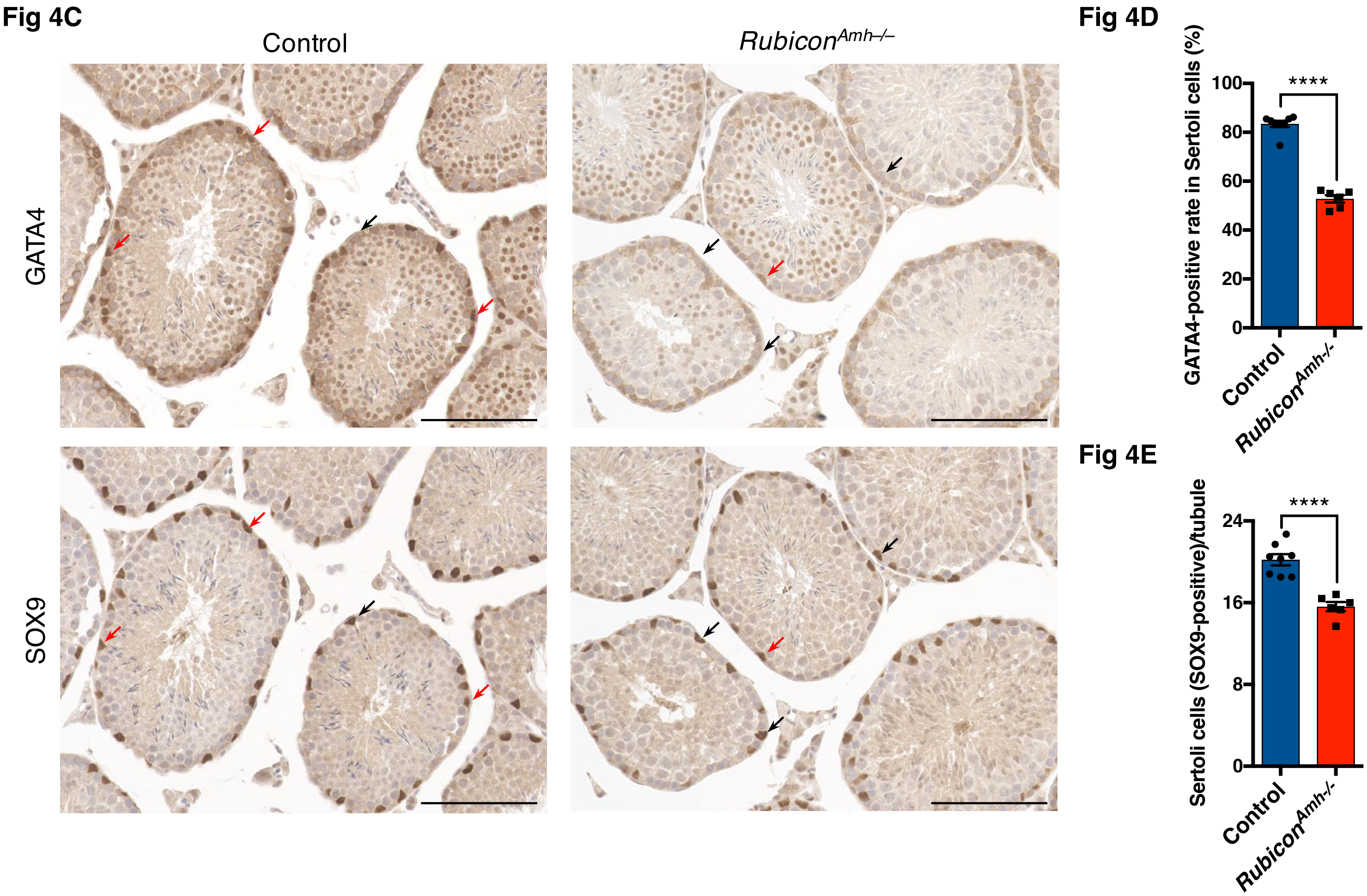

Comment-5

A direct transcriptome and/or proteome compare of the Rubicon KO Sertoli cells should be very helpful to this story.

Reply-5

Because transcriptomic or proteomic analysis requires much time and money, we additionally performed qRT-PCR assay to examine the mRNA levels of testicular genes in systemic *Rubicon* KO mice. We found that systemic loss of Rubicon did not significantly affect the mRNA levels of most of testicular genes (Figs S3A–S3F), but reduced those of Sertoli-cell-related genes (Fig 3A). This is consistent with the Reply-4 result showing that the Sertoli cell number was significantly reduced in Sertoli-cell-specific *Rubicon* KO mice (Fig 4D). These results indicate that Rubicon maintains Sertoli cell homeostasis.

Fig S3A

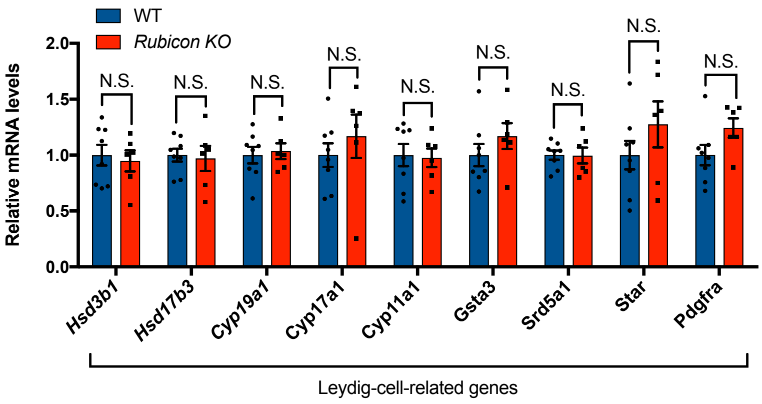

Fig S3B

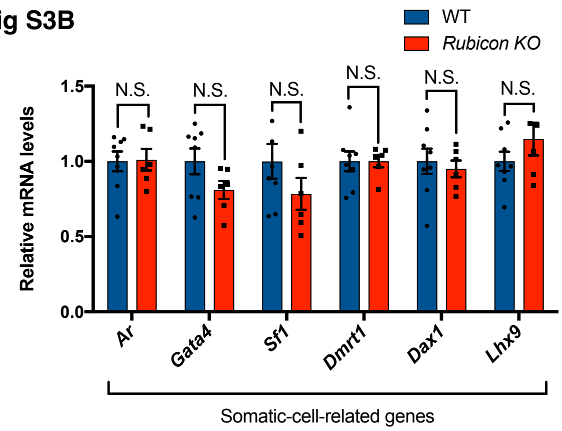

Fig S3C

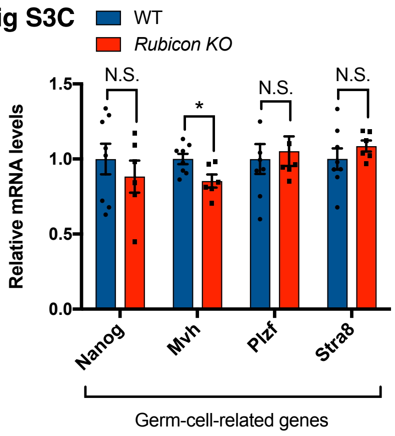

Fig S3D

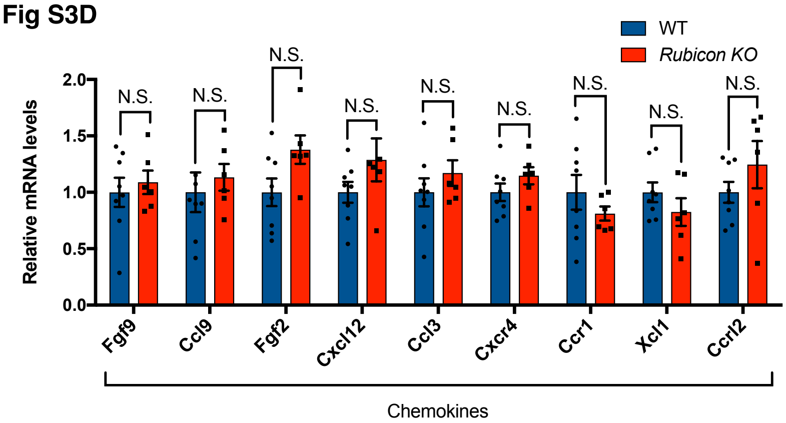

Fig S3E

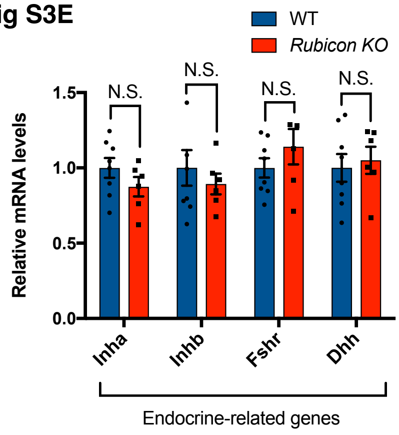

Fig S3F

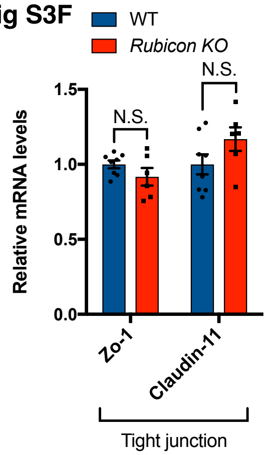

Fig 3A

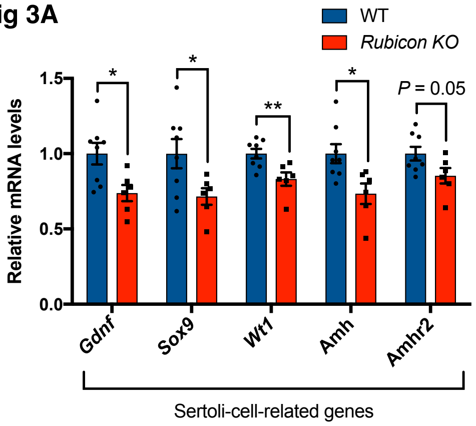

Fig 4D

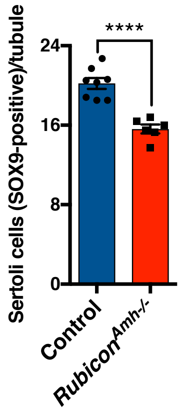

Comment-6

Sounds the text need to be further polished by native English speakers.

Reply-6

We appreciate your comment. Actually, the previous manuscript had been polished by the two dedicated native English speakers. In addition, we carefully checked the present manuscript for English grammar again.

Reviewer #2:

General comments

In this manuscript, Yamamuro and colleagues examine the role of Rubicon, a regulator of autophagy, in testicular function, focusing on GATA4 and androgens. The authors assess systemic, germ-cell-specific, and Sertoli-specific Rubicon KO mice, and find several defects in spermatogenesis, including reduced testicular weight, impaired sperm parameters, and defective seminiferous tubules in the adult testis. In general, they find that Sertoli cells are the cell type most affected by loss of Rubicon, and they pose the central hypothesis that Rubicon promotes Sertoli cell function by preventing autophagic degradation of GATA4 in an androgen-regulated manner.

The role of autophagy in spermatogenesis is not completely understood, so this study addresses a knowledge gap that is of potential interest to the field. However, there is a considerable lack of rigor in this study, and there are numerous technical issues in the assays performed. While the data supported the role of Rubicon in regulating autophagy, the specificity of autophagy for regulating GATA4 function and for androgens regulating Rubicon were less well-supported by data. Overall, the model regarding autophagy's specific function in regulating GATA4 and the role of androgens in this process is not convincingly supported. The authors should consider the following points:

Thank you so much for the valuable comments. According to your suggestions, we have carefully planned and conducted several experiments to clarify the concerns and improve the manuscript. Following is the detailed response to the comments. We have highlighted the changes in the revised manuscript using blue color.

Comment

1. There appears to be some discrepancy in the data in Figure 1. It looks like a majority of tubules are irregular in the histological image in Figure 1B, but Figure 1C shows that about only about 10% of tubules are "defective." What is the definition of defective tubules? This classification is not clearly defined. Also, what is the definition of "impaired spermatogenesis" on line 103? Also, are the images in Figure 1 representative? It looks like a few KO testes are severely reduced in testis weight, while a majority are in the range of controls; do the images in 1B correspond to those outliers?

Reply-1

We appreciate your comment. We realized that our previous results and manuscript were confusing. According to a previous report (MS Dai *et al.*, ***Andrology*** 2017), we had defined defective tubules as loss of germ cells along a significant portion of the seminiferous epithelium, germ cell sloughing, presence of large vacuoles, and tubular dilation. We used only "defective tubules" but not "impaired spermatogenesis" in the revised manuscript (Page 5, Line 93; Page 6, 111; Page 7, Line 146; Page 18, Line 400), and added the definition of "defective tubules" to Materials and Methods (Page 18, Line 399–402).

As you stated, the KO testes weights in the previous Figure 1A included severely reduced ones. Although the Figure 1B images in the previous manuscript did not correspond to these outliers, we agree with the idea that the previous Figure 1B images were overstating. Therefore, we replaced them to images that show a milder phenotype in KO testis (Fig 1B). For reference, the previous image for KO testis was shown in Fig S1B in the revised manuscript. In addition, the above outliers showed a much more severe phenotype in testis histology (Reviewer only Fig. 1). We regard this as a possibly misleading result; however, we will include it to our manuscript if you need.

Fig 1B

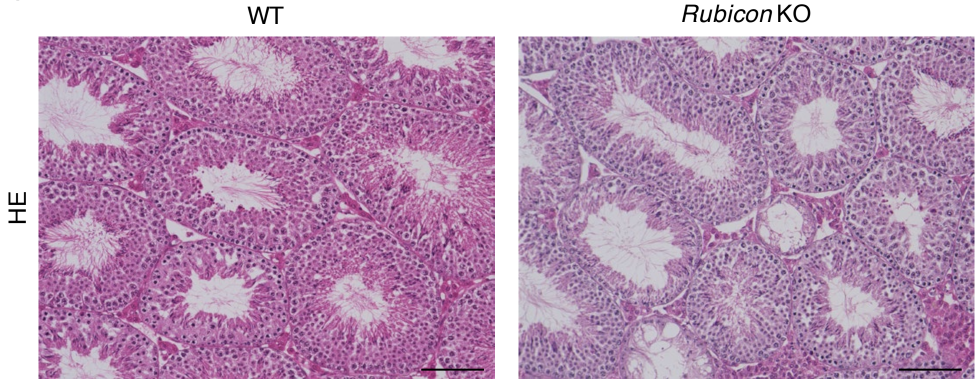

Fig S1B

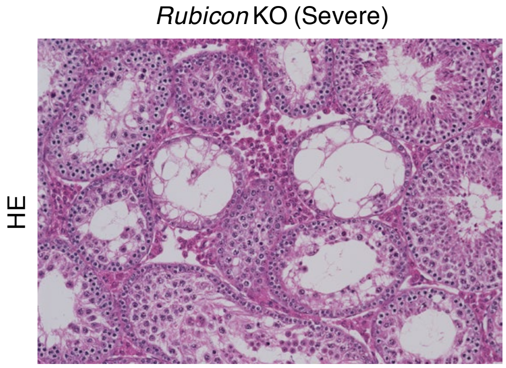

Reviewer only Fig 1

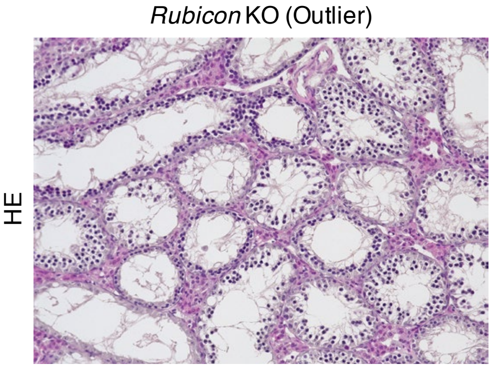

Comment

2. In Figure 1, is there any data about sperm count? In mice, sperm count could be significantly affected without significant loss in fertility as measured by pups produced per litter. Also, the pups per litter quantification in Figure 1I may be statistically significant, but is not particularly biologically compelling, since 5-6 mutant outlier litters are likely driving the statistical difference. This issue is similar to the data in 1A, where a few outliers are present and disproportionately influence the data and interpretation.

Reply-2

We did not have any data about sperm count; therefore, we examined whether systemic *Rubicon* knockout reduces sperm count, and found that the knockout did not affect sperm count (Fig S1C). Because the knockout reduced sperm motility (Figs 1F–1H) and male fertility (Figs 1I and 1J), these results indicate that the reduced male fertility could arise from a decrease in sperm motility but not in sperm count.

Thank you for clarifying a possible concern regarding our study. The five knockout male mice used in Figure 1I did not include any outlier whose testes weight is around 0.05g. As mentioned also in Reply-4, a few pups per litter was not derived from a particular male mouse (Reviewer only Fig 2). Therefore, we conclude that *Rubicon* deletion definitely reduces male fertility in mice.

Fig S1C

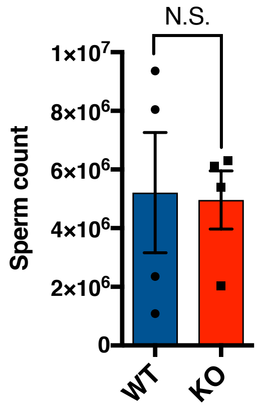

Fig 1F

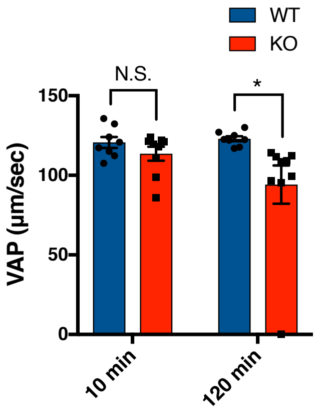

Fig 1G

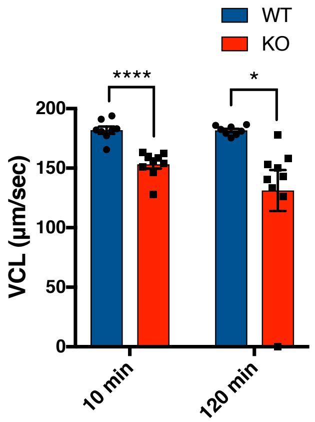

Fig 1H

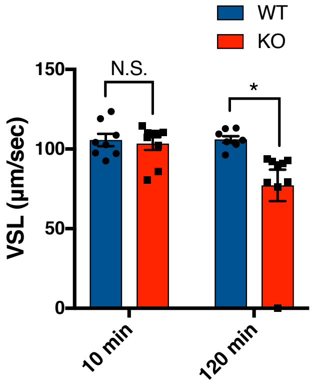

Fig 1I

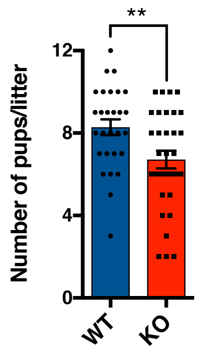

Fig 1J

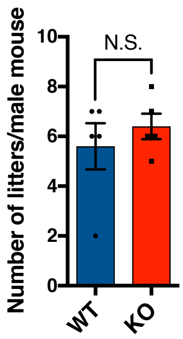

Reviewer only Fig 2

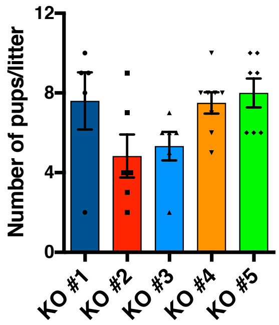

Comment

3. Related to the previous 2 points, can the authors speculate on why there are such drastic outliers in the mutant population that are significantly different? Is there any evidence of mosaicism, incomplete deletion of Rubicon, or some effects of potential remnant protein having neomorphic function?

Reply-3

While we found some outliers with a severe testicular defect in systemic *Rubicon* knockout mice, we have not found such outlier in Sertoli-cell-specific *Rubicon* knockout mice. Therefore, we speculate that *Rubicon* deletion(s) in other cell types than germ cells or Sertoli cells, such as neurons, are involved with the appearance of such outliers via unknown mechanism. For example, one or more neuronal or hormonal changes, which are also affected with growing environment, might lead to appearance of outliers. We are afraid that this speculation could be too preliminary; thus, we exclude it to the revised manuscript. If you need, we will include it to our manuscript.

Comment

4. Whereas the testis weight data in Figure 1A is based on a large number of animals (n=19-20), the breeding data in Figure 1I is based on n=5 animals. Are the smaller litters in the mutant population all linked to the same individual animal? If so, that situation would likely decrease the statistical difference between the populations.

Reply-4

As mentioned also in Reply-2, the smaller litters were not derived from one male *Rubicon* knockout mouse (Reviewer only Fig 2).

Reviewer only Fig 2

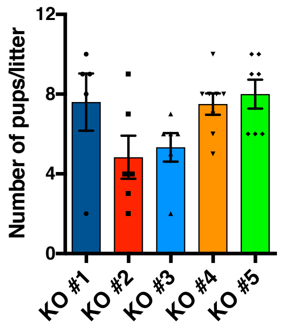

Comment

5. The choice of dataset for the scRNA-Seq analyses in Figure 2 and Figure S2 is likely not optimal. That particular study focused mainly on germ cells, and there were relatively very few somatic cells included. Using other already-published scRNA-Seq datasets (e.g., Hammoud lab, Cairns lab, and others) that include a much larger somatic component in the analysis would be much more informative. Additional violin plots for the different specific cell types would also be helpful to assess relative levels of their Rubicon expression.

Reply-5

Thank you for insightful suggestions. We got another published mouse scRNA-seq datasets that include larger somatic components (KJ Grive *et al*., ***PLOS Genetics*** 2019), and asked a bioinformatician to reanalyze the datasets. As well as the previous scRNA-seq analysis, the additional violin plots showed that the mRNA levels of Rubicon were ubiquitous, and higher in spermatocytes in mice (Figs 2B, 2C, and S2A).

Fig 2B

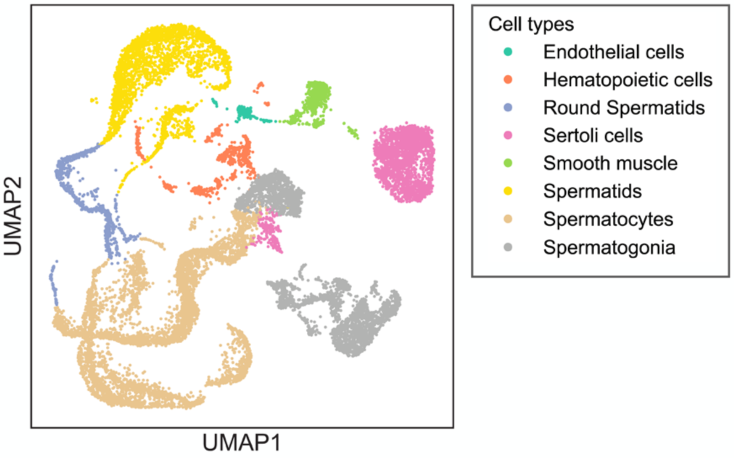

Fig 2C

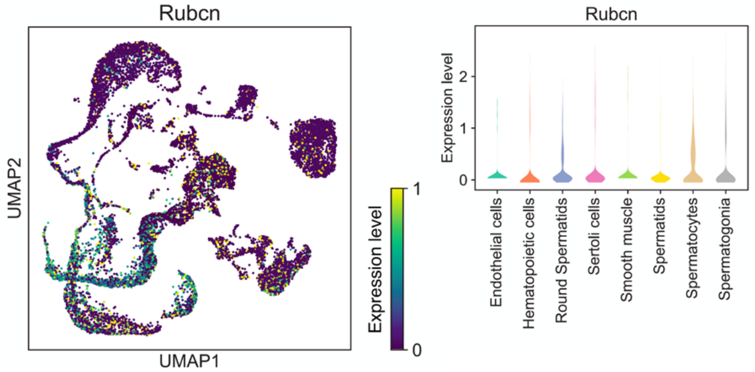

Fig S2A

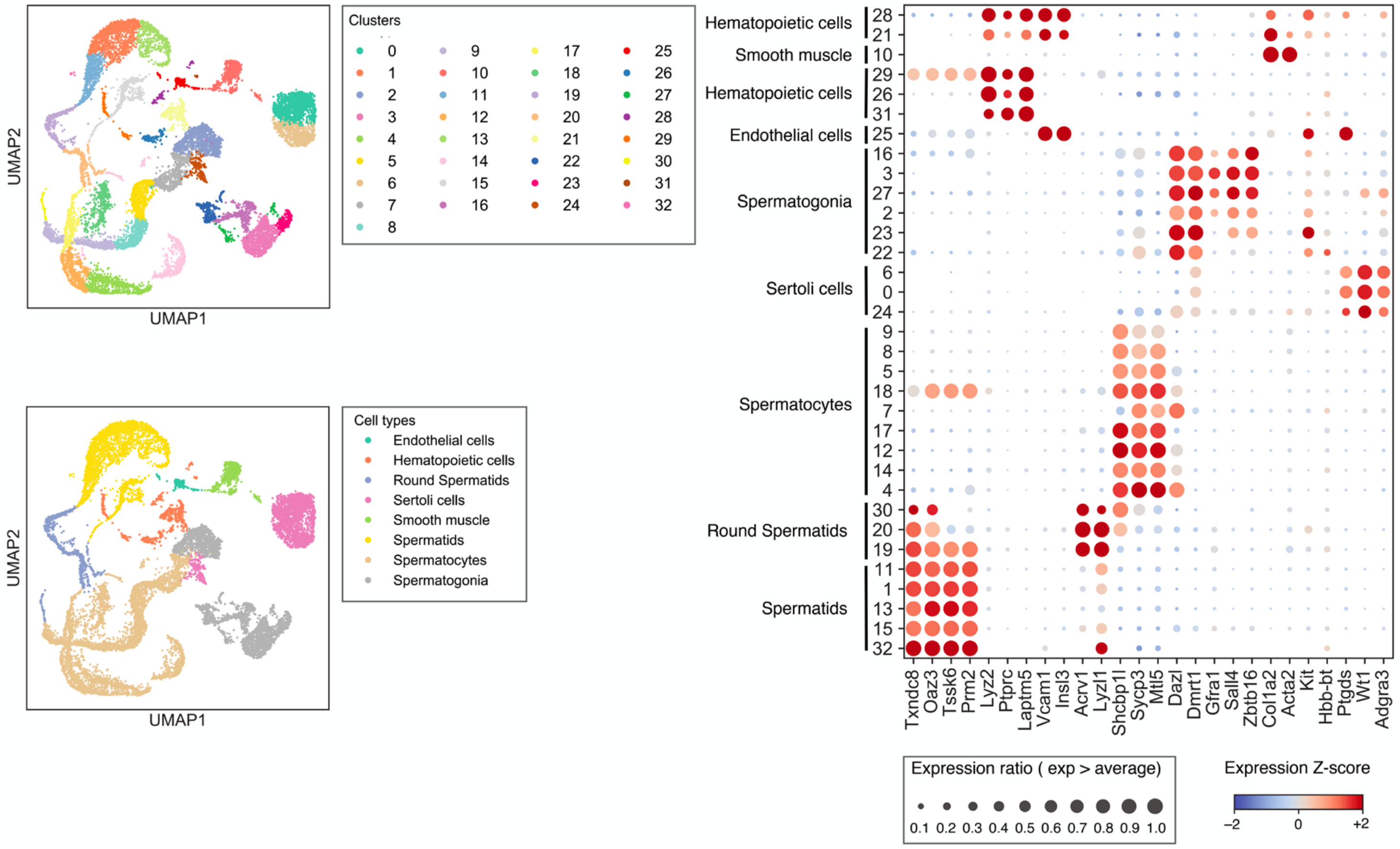

Comment

6. In Figure 2C, an additional lower-magnification image would be informative. Also, a mock-injected W testis or uninjected W testis should be included as controls in both the images and quantification.

Reply-6

According to your comment, we added a lower-magnification images to the previous Fig 2C (the present Fig 2D). Because a previous report showed that spermatogenesis is lost in an uninjected W testis (T Ogawa *et al*., ***Nat Med.*** 2000), we did not make a mock-injected or uninjected W testis sample. We added the associated references to the revised manuscript.

Fig 2D

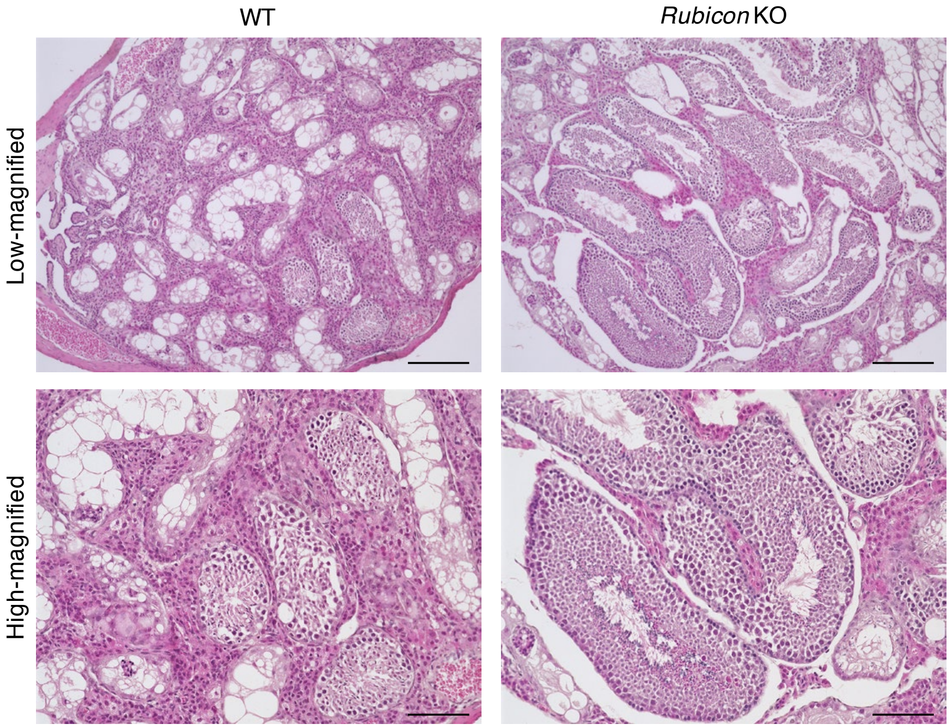

Comment

7. Is there a reason for why testis weight in Figure 1 was displayed as total of both testes (~200g) versus single testis weight (~100g) in Figure 3? These measurements should be consistent throughout.

Reply-7

We agree with the idea that these measurements should be consistent and be displayed as single testis weight. Because we had not quantified the single testis weight of systemic *Rubicon* knockout mice, we requantified single testis weight of the systemic knockout mice in the revised Figure 1A, as well as Figure 3B. The revised version of Figure 1A did not include any outlier. If you need, we will replace the Figure 1A to the previous one again.

Fig 1A

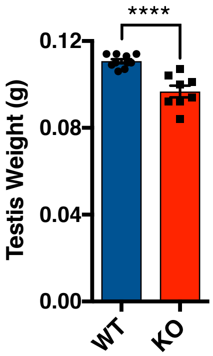

Comment

8. Is there a quantitative reduction in Sertoli cell number in Rubicon KO versus controls? This would address the findings in Figure 3A, in which reduced Sertoli cell gene expression could be caused by either a loss of Sertoli cells or reduction of gene expression within a normal number of Sertoli cells. A reduced number of Sertoli cells would also help account for a reduction in number of undifferentiated spermatogonia, since Sertoli cell number is a limiting factor in how many SSCs can be housed in the testis. The histological images and staining in Figure 4C are of poor quality and is difficult to see the Sertoli cells clearly. These images should be improved, and the appropriate quantifications should be performed.

Reply-8

This is a very important point for our study. To clarify whether Sertoli cell number is reduced in Sertoli-cell-specific *Rubicon* knockout mice, we performed immunostaining for SOX9. The SOX9-positive Sertoli cells were substantially decreased in the knockout mice (Figs 4C and 4D), suggesting that the reduced SSC number could arise from the decrease in Sertoli cell number. This is consistent with the result showing that the mRNA levels of Sertoli-cell-related genes were reduced in systemic knockout mice (Fig 3A). We also stained the adjacent testis sections for GATA4, and quantified the GATA4-positive rate in SOX9-positive Sertoli cells (Figs 4C and 4E). The GATA4-positive rate was clearly decreased in Sertoli cells in the knockout mice, suggesting that Rubicon maintains the levels of GATA4 in Sertoli cells.

Fig 3A

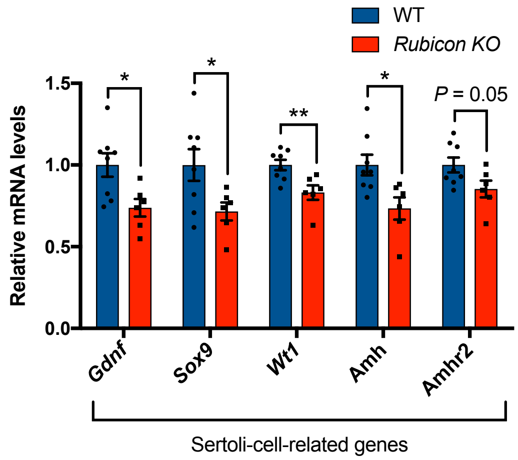

Fig 4C

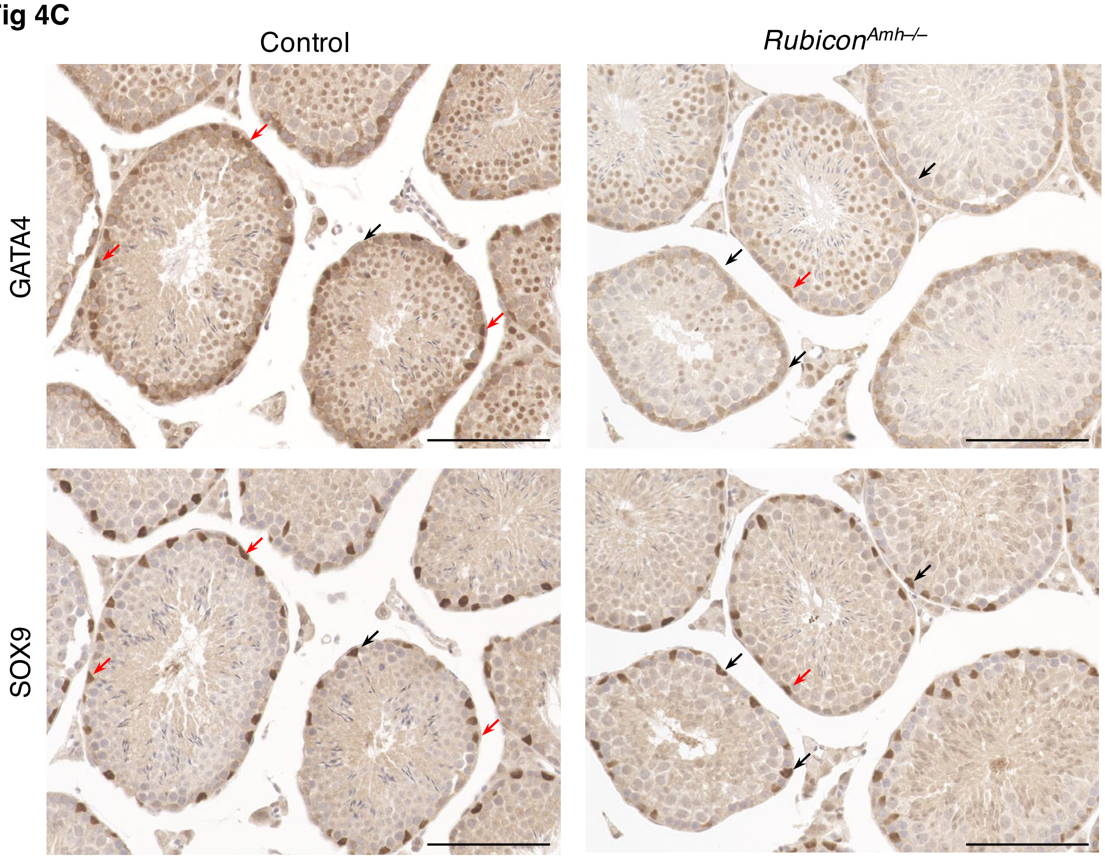

Fig 4D

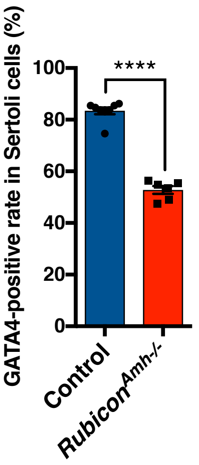

Fig 4E

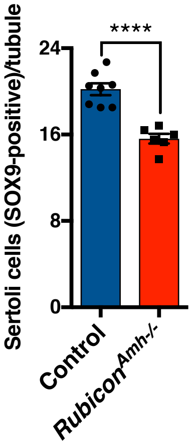

Comment

9. Is there a possibility that there is mosaicism of gene deletion in the Amh-Cre cKO model? Such a situation could potentially explain the mixed phenotype of a defective and normal tubules. A whole-testis Western does not definitively address the possibility of mosaicism and merely shows that a certain percentage of cells lost the protein. A more definitive and cell-type-specific assay (in situ? Immunostaining?) would be very informative.

Reply-9

We appreciate your constructive suggestion. To test whether there is mosaicism of gene deletion in the Amh-Cre cKO mice, we bought a Cre antibody (Sigma Aldrich, 69050) and performed its immunostaining in these mice. However, this antibody showed non-specific staining in any antigen retrieval methods including Citrate buffer, Tris-EDTA buffer or Proteinase K. Therefore, we could not test the above possibility. Considering that systemic *Rubicon* knockout mice also exhibit the mixed phenotype of defective and normal tubules, it is conceivable that the mixed phenotype is not due to potential mosaicism of gene deletion.

Comment

10. In Figure 3A, Gdnf is not a Sertoli-cell-specific gene (it is also expressed in peritubular myoid cells, as shown by Mitch Eddy's lab) and Ar is not a Leydig-cell-specific marker, since it is robustly expressed in Sertoli cell nuclei in the adult testis. Gata4 and Sf1 (Nr5a1) are also not Sertoli-cell-specific, since they are also expressed in interstitial cells such as Leydig cells. Overall, Figure 3A is not very informative for these reasons.

Reply-10

Thank you for the constructive comment. We have realized that the previous Fig 3A was not very informative. In the present Figure 3A, we showed the mRNA levels of *Gdnf*, *Sox9*, *Wt1*, *Amh*, and *Amhr2* as "Sertoli-cell-related" genes but not as "Sertoli-cell-specific" genes. The mRNA levels of other somatic-cell-related genes or Leydig-cell-related were presented in Figures S3A and S3B.

Fig 3A

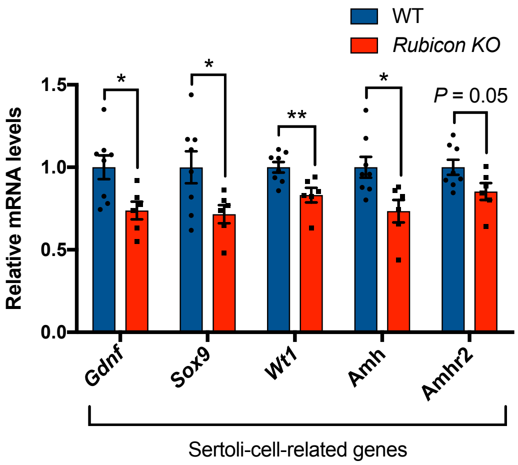

Fig S3A

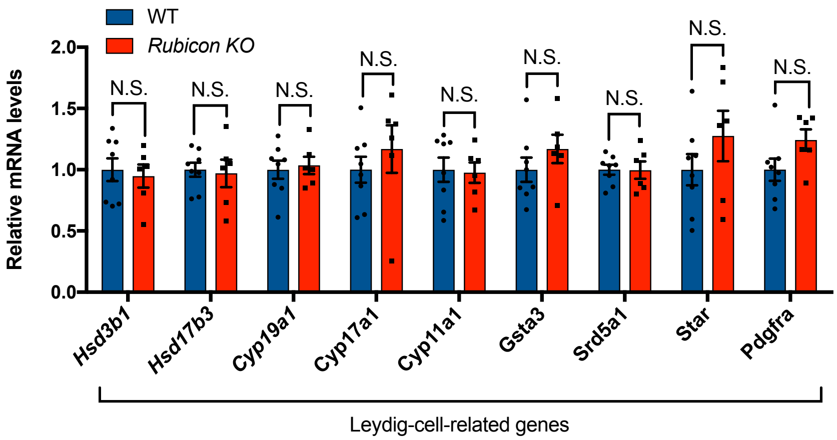

Fig S3B

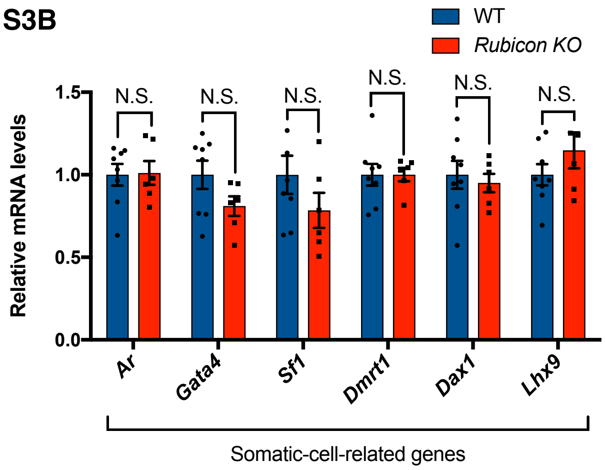

Comment

11. Can some ANOVA or multi-sample statistical comparison be done between the systemic KO and Sertoli-specific conditional KO to determine if drop in testicular weight, etc., in systemic KO mice is completely accounted for by its loss in Sertoli cells? That would eliminate the possibility that Rubicon has any function in other somatic cells.

Reply-11

Due to statistical reasons, we could not compare the phenotypes of both knockout mice. Instead of this, to test whether Rubicon in Sertoli cells completely account for the phenotypes in systemic KO mice, we assessed the male fertility in Sertoli-cell-specific *Rubicon* knockout mice. As well as the whole-body knockout, *Rubicon* knockout in Sertoli cells caused a decline in the number of pups per litter but not that of litters (Figs 3E and 3F), suggesting that Rubicon in Sertoli cells is required for normal male fertility. Together with other results, at least, loss of Rubicon in Sertoli cells would be a major contributor to the testicular defects in systemic *Rubicon* knockout mice.

Fig 3E

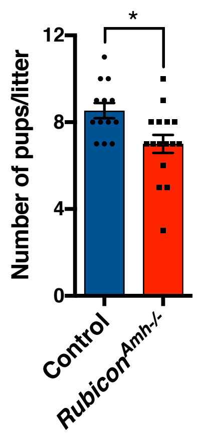

Fig 3F

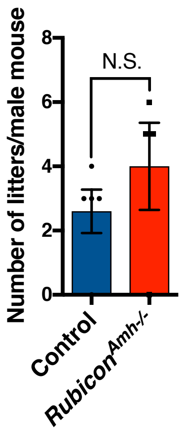

Comment

12. The analyses done for the Amh-Cre KO model in Figure 3, such as undifferentiated spermatogonia counts, should also be done for the systemic KO mice, to determine if Sertoli-specific loss of Rubicon is sufficient to account for all defects in systemic KO mice.

Reply-12

We performed immunostaining for undifferentiated spermatogonia in the systemic KO mice, and found that systemic KO mice also exhibited a reduction in the number of PLZF-positive cells (Figs S4E and S4F) and GFRA1-positive cells (Figs S4G and S4H). This result supports the idea that loss of Rubicon in Sertoli cells is sufficient to accounts for all the testicular defects in systemic KO mice.

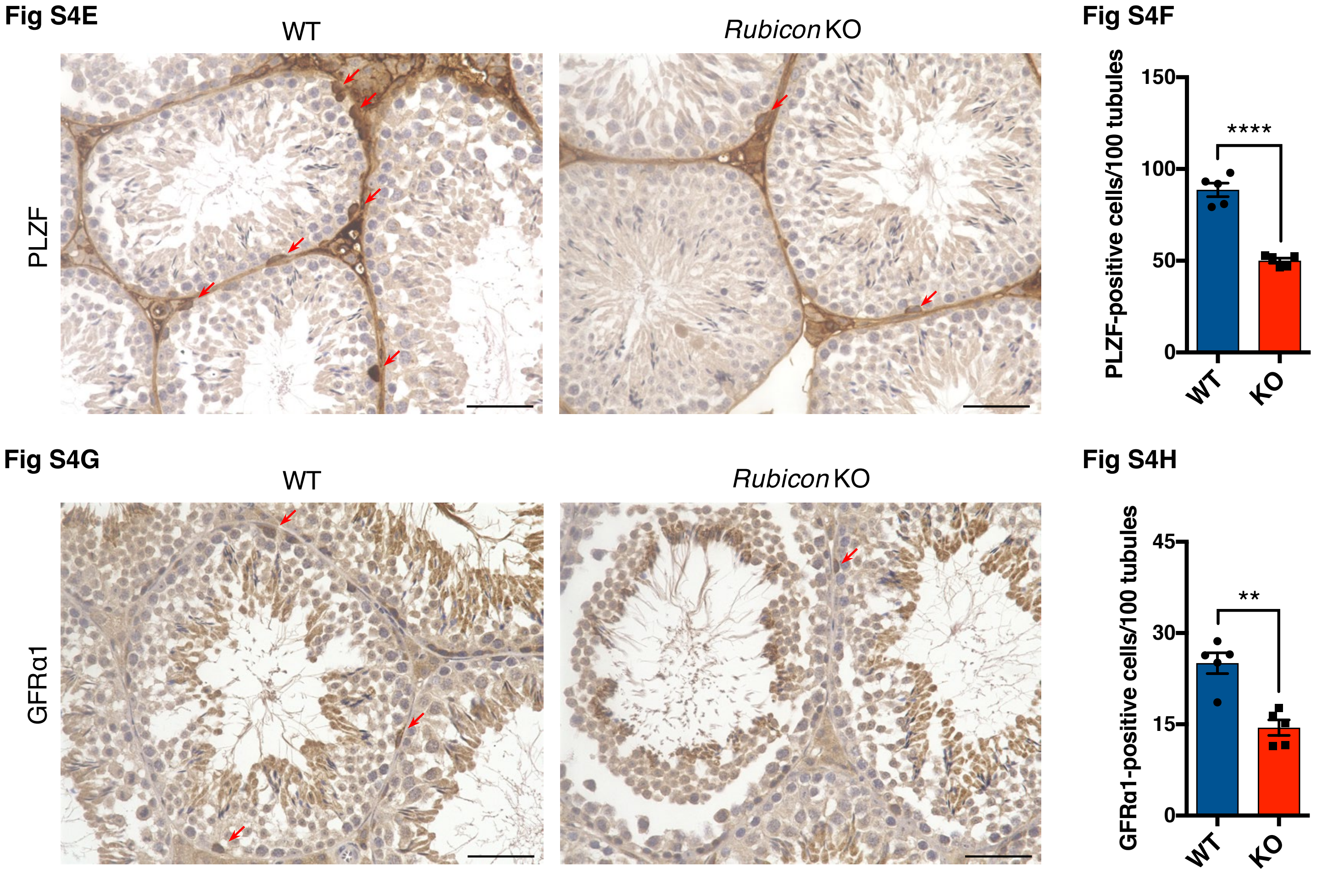

<u>Comment</u>

13. The immunostaining images for GFRA1 and PLZF in Figure 3 have a significant amount of background staining, which makes the specific cell types of interest difficult to see. Improved images would be very helpful to the reader.

<u>Reply-13</u>

Thank you for the suggestion. We previously sought to reduce a background staining for GFRA1 and PLZF, but there was a remaining background in our experiments. A similar background was seen in a previous report using the same antibodies for GFRA1 and PLZF (T Endo *et al*., ***PNAS*** 2015). To improve visibility, we replaced them with the enlarged images as more suitable images (Figs 3G–3J). We wish that you and the readers will feel them easy to see.

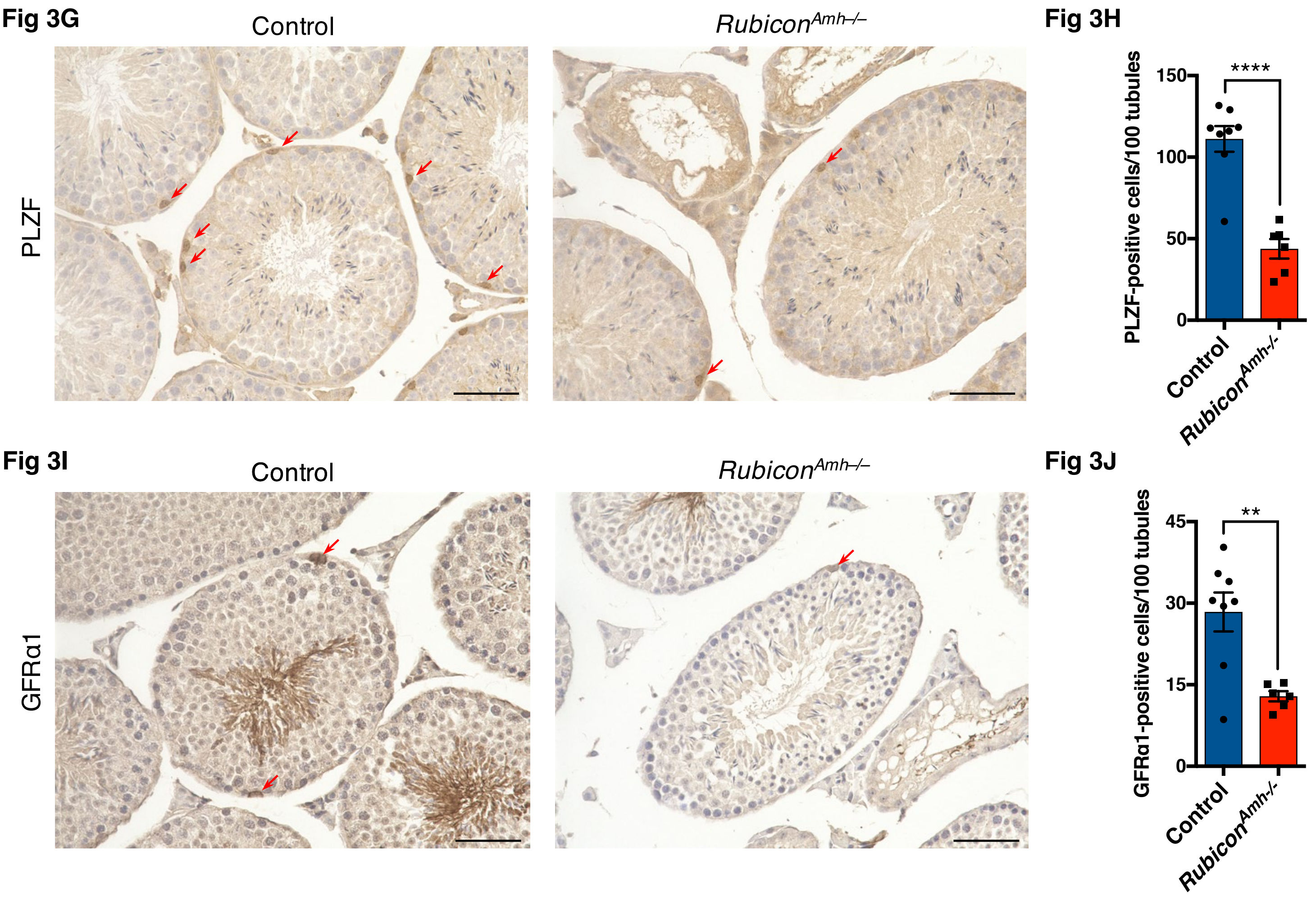

Comment

14. Given the significant defects in Sertoli cells in Rubicon KO mice, it would be more informative to assess levels of FSH, FSHR, and inhibin B rather than only looking at testosterone.

Reply-14

We measured plasma levels of FSH in systemic *Rubicon* KO mice, and found that the knockout mice exhibited comparable or possibly higher levels of FSH (Fig S4B). The mRNA levels of cytokine genes or endocrine-related genes including Fshr, Inha, or Inhb, were not significantly altered in the testes of the knockout mice (Figs S3D–S3F). These results suggest that a possible reduction of hormones or cytokines is not a major contributor to the defects in systemic *Rubicon* KO mice.

Fig S4B

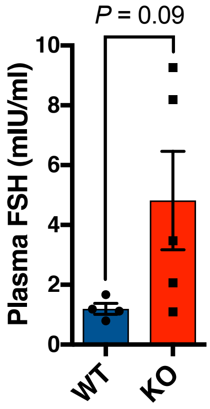

Fig S3D

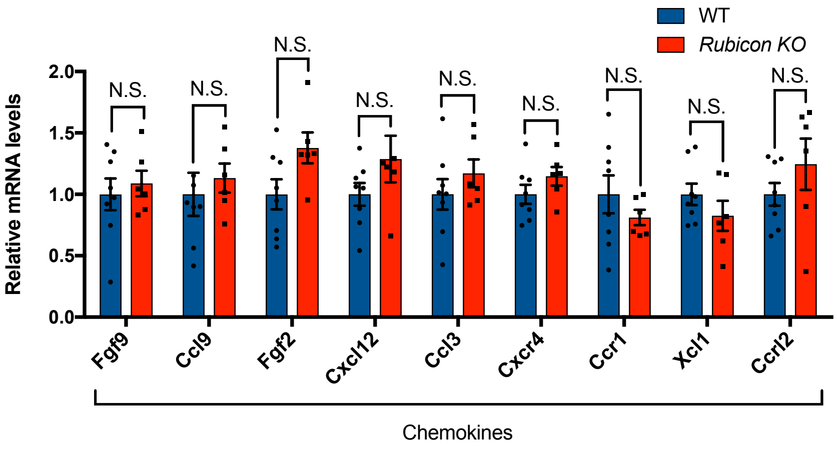

Fig S3E

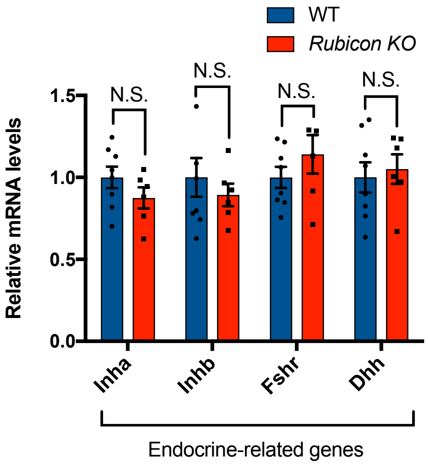

Fig S3F

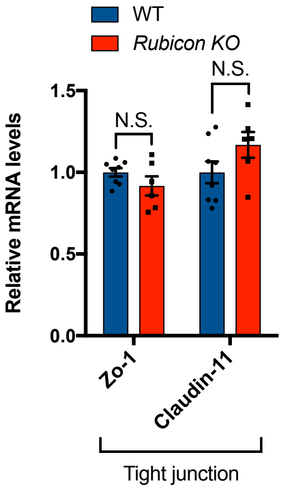

Comment

15. The authors should assess the status of the blood-testis barrier in Rubicon KO mice, given the significant impacts on the Sertoli cells. At a minimum, assessing expression levels and localization of a few BTB components would be helpful, and biotin tracer assays to test barrier function would be even more informative if possible.

Reply-15

Thank you for the insightful suggestion. To assess the status of the blood-testis barrier, we performed immunostaining for ZO-1, a tight junction protein, in the testis sections of Sertoli-cell-specific *Rubicon* knockout mice. We found that the localization of ZO-1 in the knockout mice were comparable with control mice (Fig S4D). Consistent with this, the mRNA levels of tight-junction genes were not significantly changed in systemic *Rubicon* knockout mice (Fig S3F). These observations suggest that blood-testis barrier is maintained in the knockout mice.

Fig S4D

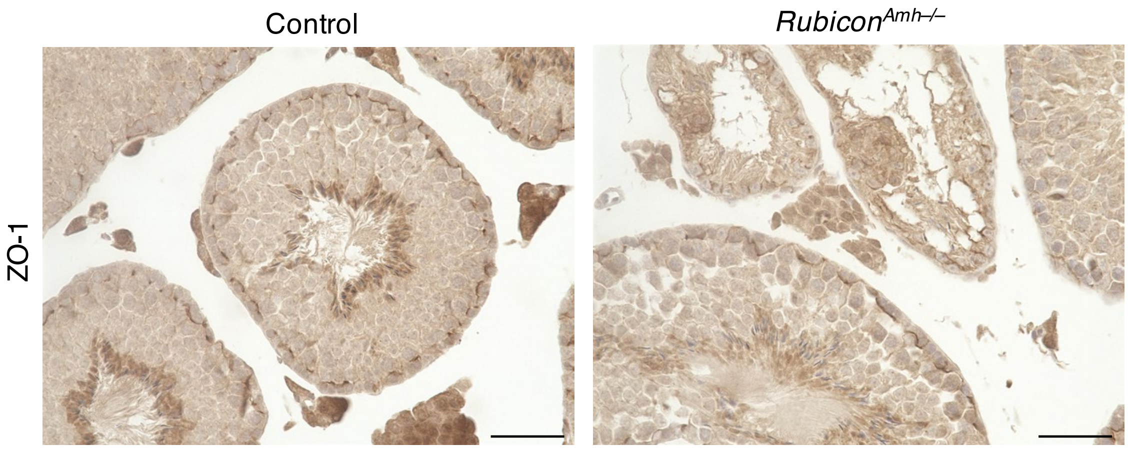

Fig S3F

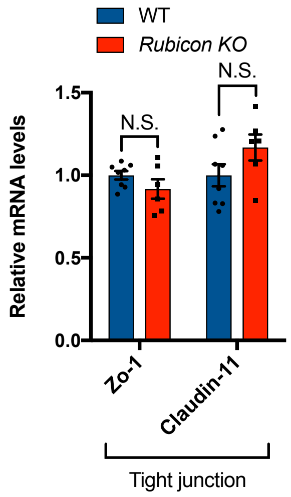

Comment

16. In Figure 4, it seems unexpected that there is more GATA4 protein in the cytoplasm than in the nucleus. One would expect that a transcription factor such as GATA4 would be highly enriched in the nucleus under normal conditions, and in immunofluorescence images of the testis, GATA4 seems to be highly enriched in the nucleus. Is there a precedent for so much GATA4 protein in the cytoplasm? One would think that if GATA4 "constitutively shuttles between the cytosol and nucleus" (line 180) to this extent, it would have already been reported in the field. These unusual results lead to some serious concerns about artefacts of using the 15-P1 cell line for these assays.

Reply-16

A previous report showed that a transcription factor GATA4 is a autophagic substrate in the context of cellular senescence (C Kang *et al*., ***Science*** 2015). Likewise, we found that GATA4 could be degraded by autophagy in Sertoli cells. Because autophagy can degrade cytoplasmic proteins but not nuclear ones, we performed cellular fractionation and found that a lysosomal inhibitor increased the cytoplasmic levels of GATA4 prior to the nuclear GATA4 (Figs 5H and 5I). And importantly, a lysosomal inhibitor chloroquine clearly rescued the reduction of GATA4 in systemic *Rubicon* knockout mice (Figs 4F and 4G). Collectively, these results indicate that cytoplasmic GATA4 is degraded by autophagy. We consider that cytoplasmic GATA4 in testis exists but is undetectable in immunostaining. In the revised manuscript, we simply claimed that "cytoplasmic GATA4 is degraded by autophagy in Sertoli cells" (Page 10, Line 208–210).

Fig 5H

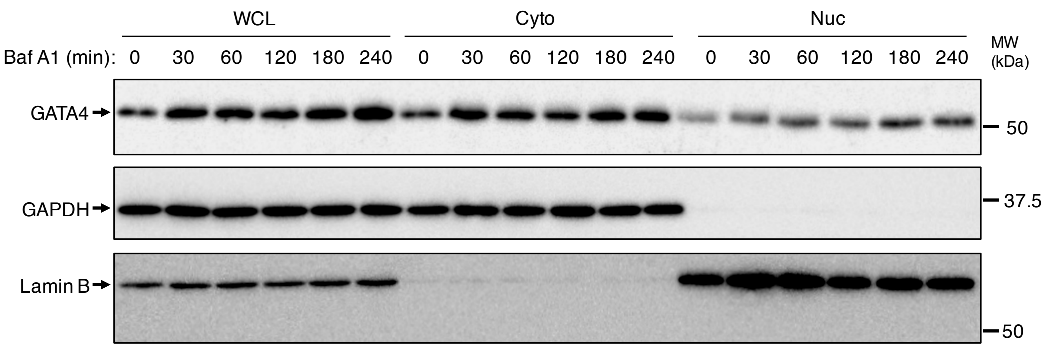

Fig 5I

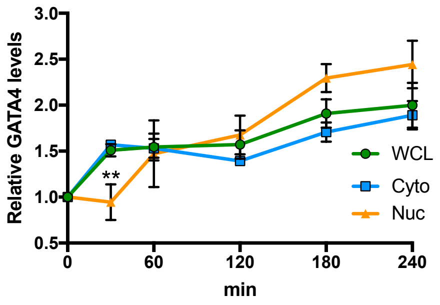

Fig 4F

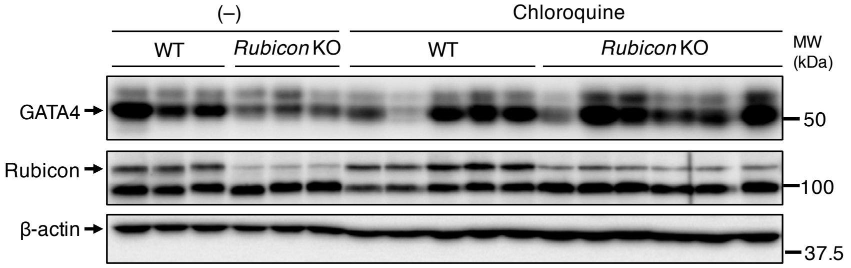

Fig 4G

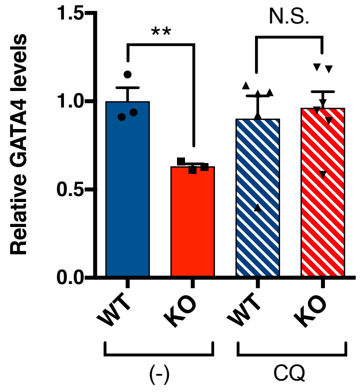

Comment

17. Are any of the results in Figure 4I statistically significant? There is no denotation of significance. If not, there is no support for any of the claims made concerning this data on lines 178-181.

Reply-17

Thank you for the comment. In the previous Fig 4I (the present Fig 5I), the GATA4 levels in the nuclear fraction was significantly lower on one-way ANOVA. We added this information to the revised manuscript.

Fig 5I

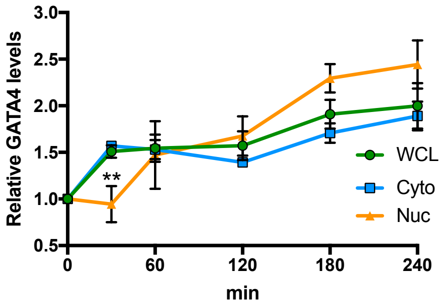

Comment

18. Do 15-P1 cells express androgen receptor in culture? This should be mentioned. Also, is the anti-androgen treatment specific to GATA4, or is there a global disruption of Sertoli cell function, or even of general health/viability, after anti-androgen treatment?

Reply-18

15P-1 cells expressed Androgen receptor (Figs 5C, 6A, and 6H). This is mentioned in the revised manuscript (Page 10, Line 222). To assess whether anti-androgen treatment causes a global disruption of Sertoli cell function, we examined the levels of other proteins. We found that anti-androgen treatment did not altered the levels of SOX9 or AR, but decreased those of WT1 in 15P-1 cells (Figs 6A–6D). The same tendency was observed in mice treated with an androgen antagonist (Figs 7A–7D). In addition, as shown in Reply-21, GATA4 is a specific substrate for autophagy in Sertoli cells. Therefore, we consider that anti-androgen treatment is specific to GATA4 and WT1, and that the regulation of GATA4 by androgen is dependent on autophagy. The regulation mechanism of WT1 by androgen is to be examined in the future study.

Fig 5C

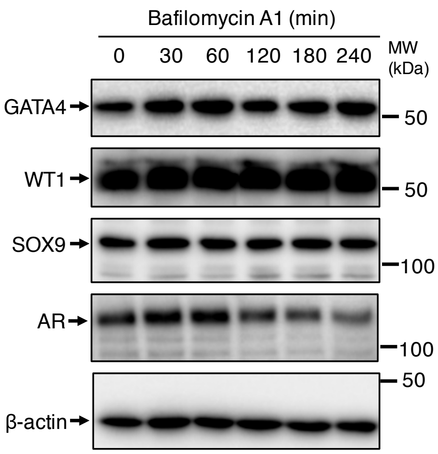

Fig 6H

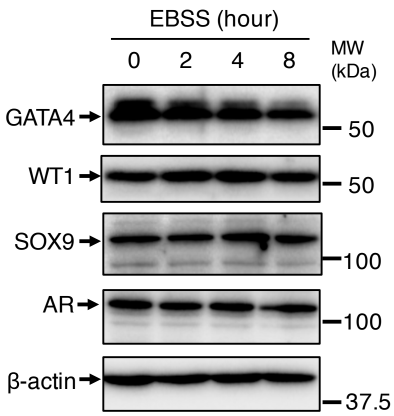

Fig 6A

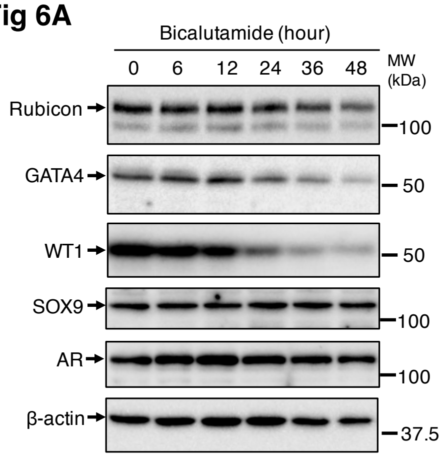

Fig 6B

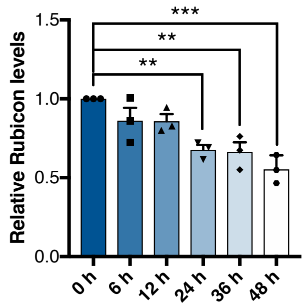

Fig 6C

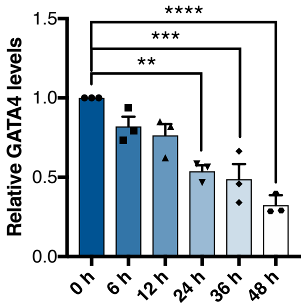

Fig 6D

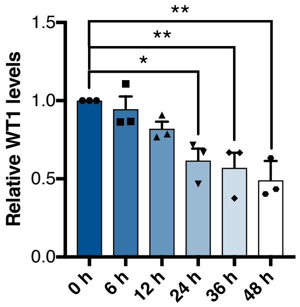

Fig 7A

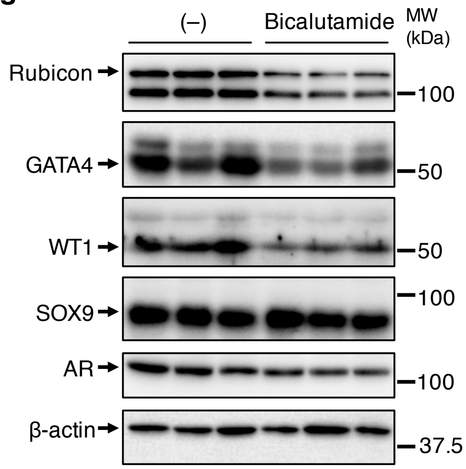

Fig 7B

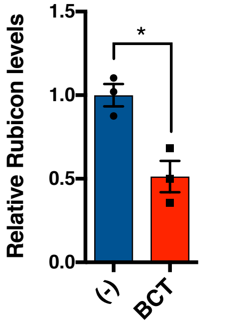

Fig 7C

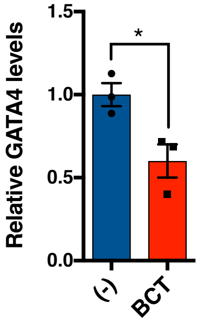

Fig 7D

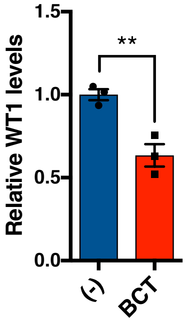

Comment

19. In general, the human samples in Figure 5 are not very convincing, and the stainings, especially for GATA4, are of poor quality. As is the case for mouse, quantification of Sertoli cells needs to be done for the human samples in Figure 5.

Reply-19

Because the sections of the clinical samples are very limited, we redid immunostaining only for GATA4, and quantified the number of GATA4-positive cells. The GATA4-positive cells were significantly reduced in the patient receiving anti-androgen therapy (Figs 7F and 7G). Because we did not quantify the GATA4-positive rate in Sertoli cells, we simply claimed that the anti-androgen therapy caused a reduction of GATA4 in testis, but not in Sertoli cells (Page 11, Line 233–237).

Fig 7F

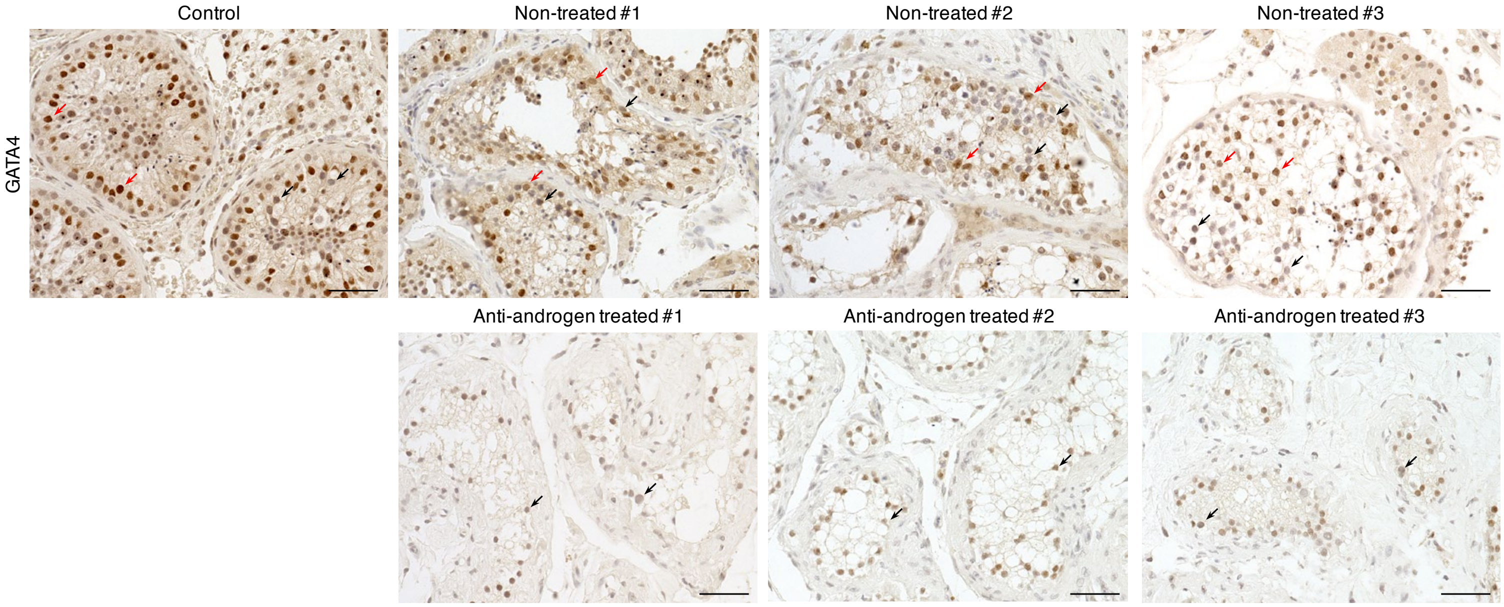

Fig 7G

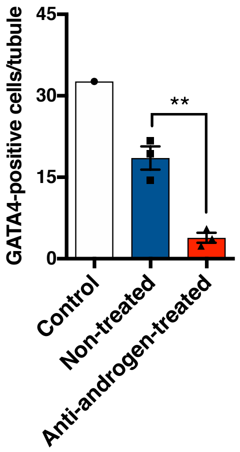

Comment

20. Overall, the claim that “androgen maintains the testicular levels of Rubicon, thereby maintaining adequate levels of GATA4 protein in Sertoli cells” (lines 203-204) is not convincingly supported by the data, especially with a lack of in vivo data regarding this point. The human data is correlative and or poor rigor, and the mouse data is based on an in vitro model that is potentially questionable in its representation of Sertoli cells in vivo.

Reply-20

We appreciate your insightful comment. According to your comment, we injected mice with an androgen antagonist, and found that anti-androgen treatment caused a significant decrease in the testicular levels of Rubicon and GATA4 in mice (Figs 7A–7C). Together with the human data and the *in vitro* data, we simply claimed that androgen maintains the testicular levels of Rubicon and GATA4 in mouse and human. And we removed “in Sertoli cells” from the previous lines 203-204 (Page 11, Line 236, 237).

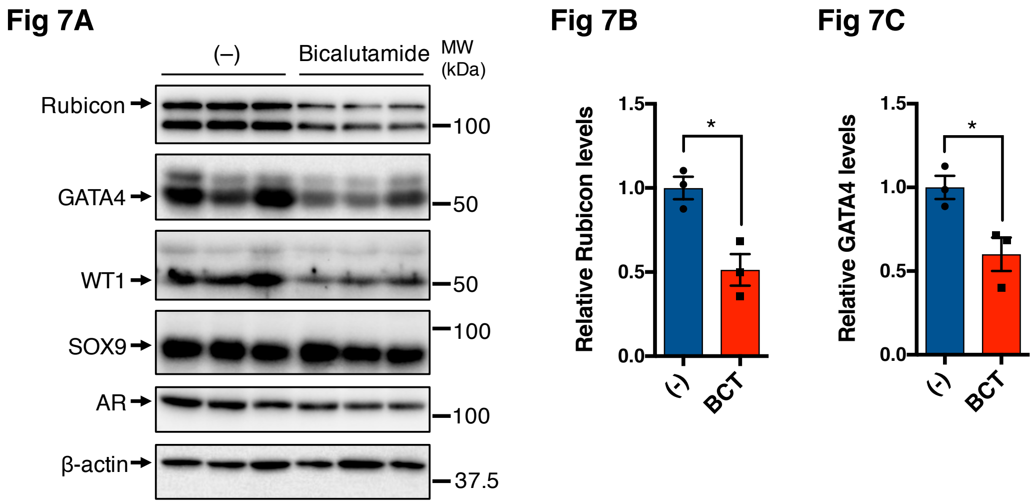

Comment

21. Is bafilomycin A1 specific to GATA4? Or does it affect a number of Sertoli-cell-specific proteins (or all proteins in general) that could influence Sertoli cell function? In other words, is autophagy a truly specific regulator of GATA4? Similarly, does anti-androgen treatment in Figure 5 also specifically affect GATA4, or is it a general disruption of Sertoli cell proteins and function? These scenarios are not definitively addressed in this study.

Reply-21

A lysosomal inhibitor bafilomycin A1 increased the levels of GATA4 but not WT1, SOX9, and AR in 15P-1 cells (Figs 5C–5E). In addition, the levels of GATA4 was specifically reduced in a starvation medium EBSS, in which autophagy is strongly induced (Figs 6H–6J). These results indicate that autophagy is a truly specific regulator of GATA4 in Sertoli cells. Anti-androgen treatment reduced both GATA4 and WT1, but not SOX9 or AR, in 15P-1 cells (Figs 6A–6D) and in mice (Figs 7A–7D). Hence, it is conceivable that there is a specific regulation of GATA4 or WT1 by androgen. We consider that the regulation of GATA4 by androgen is dependent on autophagy.

Fig 5C

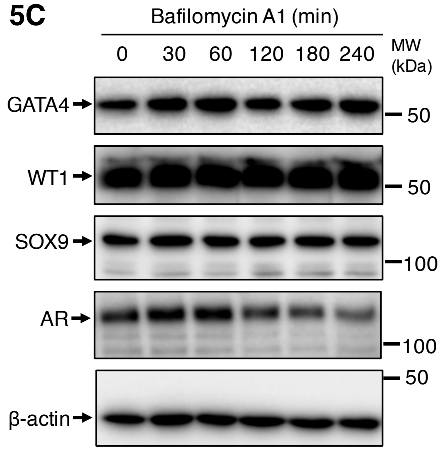

Fig 5D

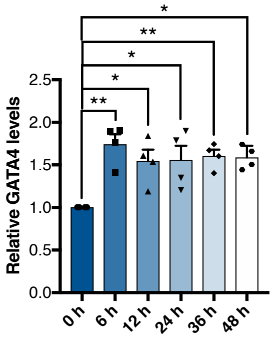

Fig 5E

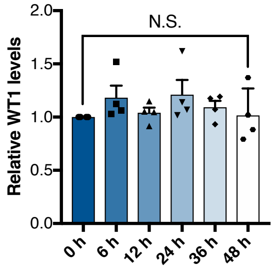

Fig 6H

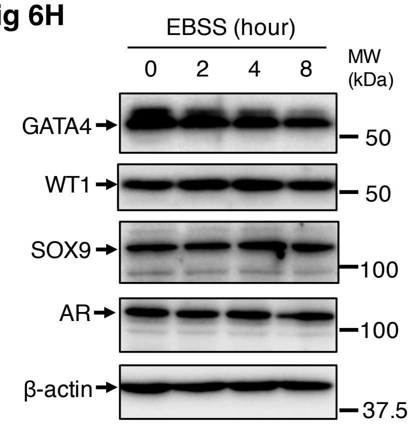

Fig 6I

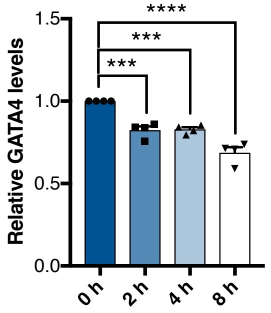

Fig 6J

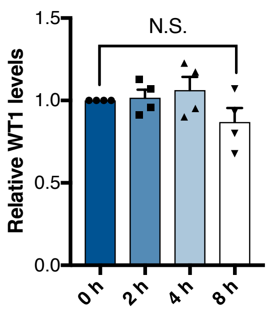

Fig 6A

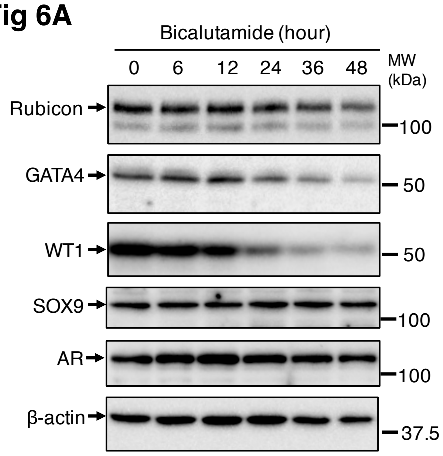

Fig 6B

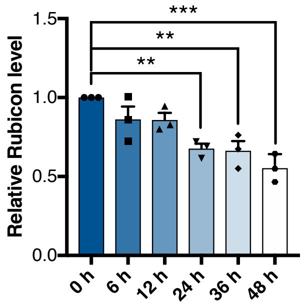

Fig 6C

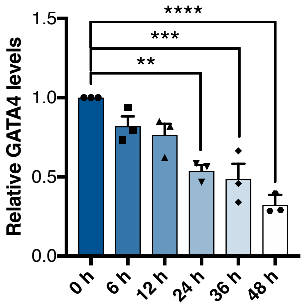

Fig 6D

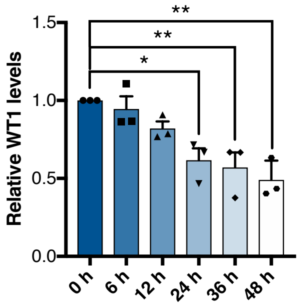

Fig 7A

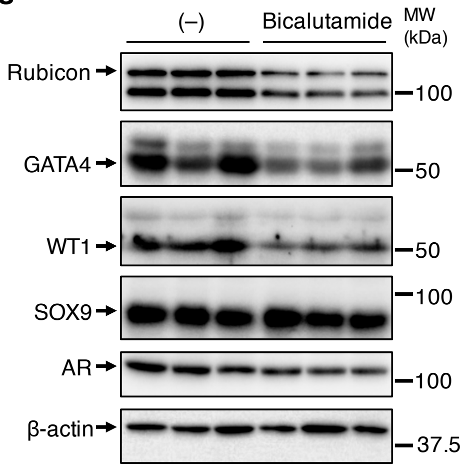

Fig 7B

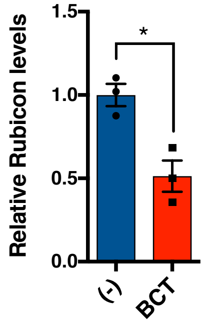

Fig 7C

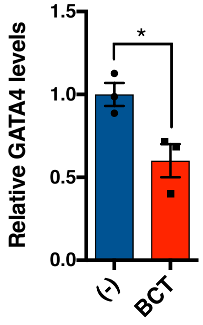

Fig 7D

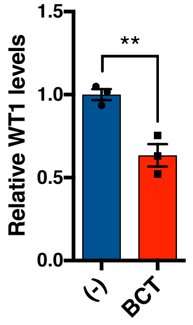

Comment

22. More details are needed for the transplantation assays in the Methods section. How many cells were injected? How were the cells prepared? Was there any enrichment for spermatogonia/SSCs? Also, usually the read-out of these assays is the number of spermatogenic colonies formed within whole-mount tubules, but I suppose that would require a lacZ or fluorescent label present to label the donor cells versus the host cells.

Reply-22

4×10^6 cells/testis were injected. We prepared the donor cells according to a previous report (T Ogawa *et al*., ***Nat Med.*** 1997). We did not enrich SSCs. We have added the detailed information of the transplantation assay (Page 15, 16, Line 340–347). We agree with the idea that it is better to label the donor cells and the host cells. However, the transplantation of unlabeled donor cells would be sufficient to examine whether *Rubicon* knockout germ cells settled at a comparable level to wild-type cells. Indeed, the knockout germ cells settled in the tubules as efficiently as wild-type cells (Fig 2D and 2E). In addition, we also found that germ-cell-specific *Rubicon* knockout mice had no defect in testis (Fig 2F). These data indicate that Rubicon in germ cells is dispensable for spermatogenesis. This is consistent with our other findings showing that Rubicon in Sertoli cells is required for normal spermatogenesis.

Fig 2D

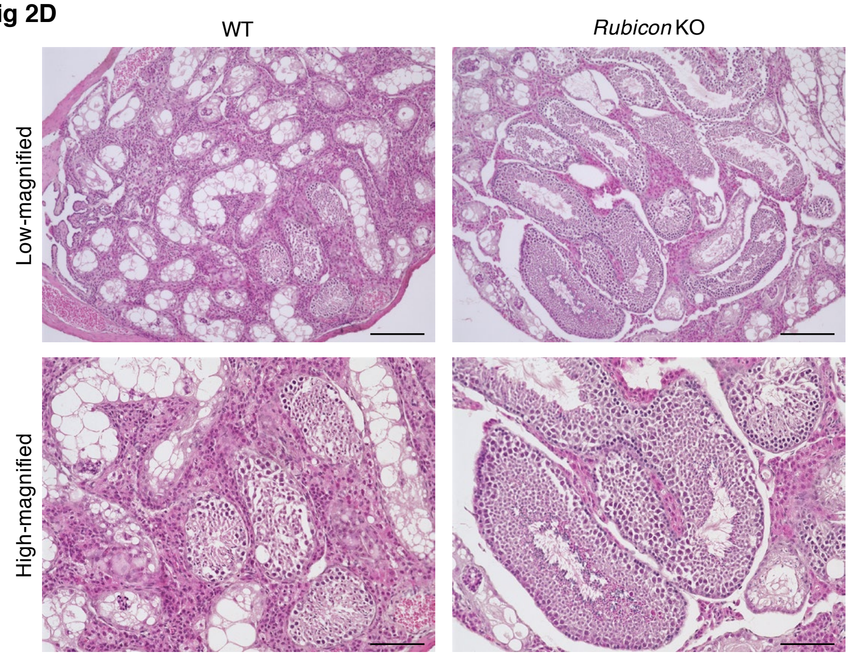

Fig 2E

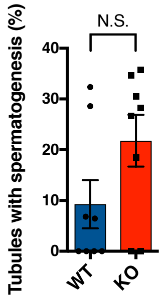

Fig 2F

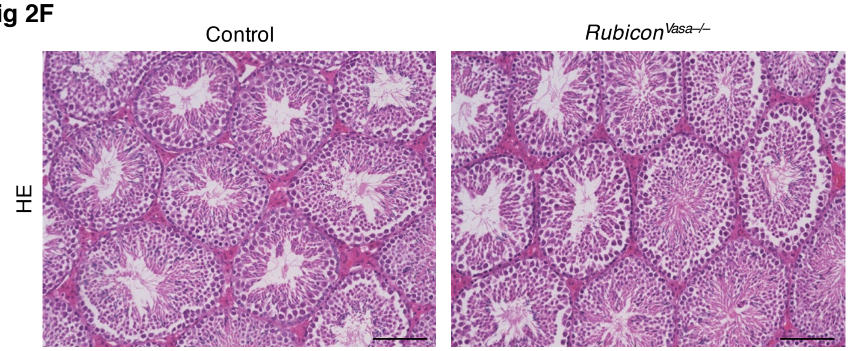

Supplement: Attachment — Submitted filename: Point-by-point response_1st_revision_5.pdf [file pgen.1009688.s010.pdf]
